# Hepatitis B virus X protein counteracts high mobility group box 1 protein-mediated epigenetic silencing of covalently closed circular DNA

**DOI:** 10.1371/journal.ppat.1010576

**Published:** 2022-06-09

**Authors:** Elena S. Kim, Jun Zhou, Hu Zhang, Alexander Marchetti, Maarten van de Klundert, Dawei Cai, Xiaoyang Yu, Bidisha Mitra, Yuanjie Liu, Mu Wang, Ulrike Protzer, Haitao Guo

**Affiliations:** 1 Cancer Virology Program, UPMC Hillman Cancer Center, and Department of Microbiology and Molecular Genetics, University of Pittsburgh School of Medicine, Pittsburgh, Pennsylvania, United States of America; 2 Department of Microbiology and Immunology, Indiana University School of Medicine, Indianapolis, Indiana, United States of America; 3 College of Life Sciences and Health, Wuhan University of Science and Technology, Wuhan, China; 4 Technical University of Munich, School of Medicine/Helmholtz Center Munich, Munich, Germany; 5 German Center for Infection Research (DZIF), partner site Munich, Munich, Germany; Pennsylvania State University College of Medicine: Penn State College of Medicine, UNITED STATES

## Abstract

Hepatitis B virus (HBV) covalently closed circular DNA (cccDNA), serving as the viral persistence form and transcription template of HBV infection, hijacks host histone and non-histone proteins to form a minichromosome and utilizes posttranslational modifications (PTMs) “histone code” for its transcriptional regulation. HBV X protein (HBx) is known as a cccDNA transcription activator. In this study we established a dual system of the inducible reporter cell lines modelling infection with wildtype (wt) and HBx-null HBV, both secreting HA-tagged HBeAg as a semi-quantitative marker for cccDNA transcription. The cccDNA-bound histone PTM profiling of wt and HBx-null systems, using chromatin immunoprecipitation coupled with quantitative PCR (ChIP-qPCR), confirmed that HBx is essential for maintenance of cccDNA at transcriptionally active state, characterized by active histone PTM markers. Differential proteomics analysis of cccDNA minichromosome established in wt and HBx-null HBV cell lines revealed group-specific hits. One of the hits in HBx-deficient condition was a non-histone host DNA-binding protein high mobility group box 1 (HMGB1). Its elevated association to HBx-null cccDNA was validated by ChIP-qPCR assay in both the HBV stable cell lines and infection systems *in vitro*. Furthermore, experimental downregulation of HMGB1 in HBx-null HBV inducible and infection models resulted in transcriptional re-activation of the cccDNA minichromosome, accompanied by a switch of the cccDNA-associated histones to euchromatic state with activating histone PTMs landscape and subsequent upregulation of cccDNA transcription. Mechanistically, HBx interacts with HMGB1 and prevents its binding to cccDNA without affecting the steady state level of HMGB1. Taken together, our results suggest that HMGB1 is a novel host restriction factor of HBV cccDNA with epigenetic silencing mechanism, which can be counteracted by viral transcription activator HBx.

## Author summary

Nowadays hepatitis B virus (HBV) prevalence remains a significant burden to the worldwide healthcare. There is no cure for chronic HBV infection so far due to the lack of therapies that enable elimination of an intrahepatic persistent form of HBV DNA genome, namely, the covalently closed circular DNA (cccDNA). Inside the infected liver cells, cccDNA hijacks host structural and regulatory factors to assemble into a minichromosome, but certain intrinsic host factors are able to counteract cccDNA activity. In our study, we identified an anti-HBV host restriction factor, specifically the high mobility group box 1 protein (HMGB1), and demonstrated its potential to mediate an epigenetic silencing, i.e. functional inactivation, of the cccDNA. However, the virally encoded accessory protein HBx is able to antagonize HMGB1 and maintain an active state of cccDNA. This may contribute to a better understanding of virus-host interaction during HBV infection, and to the development of HBV infection epigenetic drugs and re-consideration of cancer therapeutics strategies, where HMGB1 is used as an anti-cancer target.

## Introduction

Hepatitis B virus (HBV) is a hepatotropic DNA virus causing both acute and chronic infection in humans. The chronically infected liver can further develop fibrosis, cirrhosis and hepatocellular carcinoma (HCC) [1]. Chronic hepatitis B (CHB) is a global public health burden currently counting ~300 million cases worldwide, with exacerbations leading to ~1 million deaths yearly. So far, HBV vaccination is only prophylactic, and a definite cure for the CHB does not exist [2]. The chronicity of HBV infection is ultimately supported by the intracellular multicopy viral episome called covalently closed circular DNA (cccDNA), which is the *bona fide* viral transcription template resistant to all the currently available antiviral drugs [3]. Therefore, a cure of hepatitis B relies on physical eradication or permanent inactivation of cccDNA [4].

HBV is a noncytopathic, liver-tropic DNA virus belonging to the *Hepadnaviridae* family [5]. HBV life cycle requires the sodium taurocholate co-transporting polypeptide (NTCP) expressed and localized on the host cell surface serving as an entry receptor for the virion particles [6]. Upon entry, a viral 3.2kb partially double-stranded relaxed circular (rc) DNA genome is transported from nucleocapsid into the cell nucleus and converted into the episomal cccDNA using host DNA repair machinery [7–10]. cccDNA serves as a transcriptional template for the five mRNA species, including the 3.5 kb precore (pC) mRNA and pregenomic (pg) RNA, the 2.4 kb and 2.1 kb surface proteins mRNA, and the 0.7 kb HBV protein X (HBx) mRNA [11,12]. The pgRNA is reverse transcribed into rcDNA by viral polymerase inside of the cytoplasmic capsid [13,14]. Subsequently, the newly formed rcDNA-containing capsid is enveloped by viral surface proteins to form progeny virion or recycled to the nucleus to replenish the cccDNA reservoir [15,16]. Therefore, cccDNA is an essential component of the HBV life cycle and is responsible for the establishment and maintenance of viral DNA replication and gene expression.

Inside hepatocyte nucleus, HBV cccDNA exists in a form of chromatin-like structure and its transcriptional activity undergoes regulation by epigenetic mechanisms, but the detailed interactome and epigenome of cccDNA minichromosome remain elusive [9,17,18]. Growing evidence suggested that the HBV-encoded accessory protein HBx plays an indispensable role in supporting cccDNA transcription *via* maintaining a transcriptionally active epigenetic state of cccDNA [9,18,19]. HBx is a small nonstructural, regulatory viral protein of 17 kDa, which serves as a multipurpose transactivator of HBV and cellular promoters to regulate viral and host functions [20,21]. HBx activates the transcription of host genes by interacting directly with nuclear transcription factors or by activating various signal transduction pathways in the cytoplasm and has been proven to be a potent epigenetic modifying factor in the liver [20,22,23]. HBx has been shown to activate HBV transcription through its recruitment onto cccDNA by host PCAF/GCN5, p300, and CBP acetyltransferases; and to inhibit cellular factors involved in chromatin regulation, such as PP1/HDAC1 complex [24]. Conversely, in the absence of HBx, the rapid hyperacetylation of cccDNA-bound histones by p300 is severely impaired, but the deacetylases HDAC1 and SIRT1 are recruited to cccDNA, leading to the suppression of cccDNA transcription and viral replication [24]. In addition, without HBx, the silenced HBV genome is not only associated with histone hypoacetylation but also the deposition of repressive markers (H3K9me2 and H3K9me3) by methyltransferase SETDB1 [25]. A well-characterized HBx interactor is the cellular DNA damage-binding protein 1 (DDB1) [20,26–29]. Recently, the HBx-DDB1 complex was shown to recruit the cullin 4A-RING E3 ubiquitin ligase (CRL4); the assembled CRL4-DDB1 complex ubiquitinates the host structural maintenance of chromosomes protein 5/6 (Smc5/6) complex for proteasomal degradation to activate cccDNA transcription [30,31], suggesting that Smc5/6 may silence cccDNA in the absence of HBx, though it remains unknown whether the Smc5/6-mediated transcriptional inhibition of cccDNA involves epigenetic mechanisms [32]. To ensure the presence of HBx prior to Smc5/6-mediated silencing of cccDNA, HBV particles may carry HBx mRNA for the initial expression of HBx upon infection [33,34]. However, DDB1 has also been shown to stimulate HBV transcription in an HBx-independent manner [35]. Thus, it is still unclear whether the integrity of proposed HBx-DDB1-Smc5/6 axis is absolutely required for cccDNA transcription in every experimental and clinical settings.

In this study, we aimed to characterize the HBV cccDNA epigenetic landscape and epigenetic interactome to identify potential restriction host factors. To this end, we developed a dual HBV replication model cell culture system producing cccDNA in the presence and absence of HBx to recapitulate the epigenetic profiles of transcriptionally active and inactive cccDNA, respectively. Through comparative proteomic analysis of the wildtype (wt) and HBx-null cccDNA, the high mobility group box 1 protein (HMGB1) was identified as a novel epigenetic silencer of cccDNA, which may serve as target for the development of epigenetic therapies to cure CHB and prevent CHB-related liver cirrhosis and its malignant transformation to HCC.

## Results

### Effect of HBx on transcription from transiently transfected HBV genome

Among a plethora of reported functions of HBx, its key role in HBV replication cycle is to serve as a transcription transactivator of viral episomal DNA template, either the genuine cccDNA or transfected HBV plasmid [19,20,36]. To confirm that, the viral RNA transcription and core DNA replication were assessed in HepG2 cells upon wt HBV plasmid pCMVHBV transfection in comparison with HBx-null mutant pCMVHBVΔx. The results demonstrated that HBV plasmid-based viral RNA transcription and core DNA replication were dramatically reduced in the absence of HBx expression, which were restored upon HBx trans-complementation (S1A Fig). Considering that the transcription of HBV pgRNA and subgenomic RNA from pCMVHBV is governed by CMV-IE promoter and HBV surface promoters, respectively, the results indicate that the HBx-mediated enhancement of HBV transcription is independent of viral promoter, which is consistent with a previous study showing that HBx selectively stimulated gene expression from extrachromosomal DNA template [36]. Further experiments with trans-complementation of the HBx-minus system with gradually increasing HBx expression demonstrated that HBx dose-dependently enhanced HBV transcription and the subsequent DNA replication (S1B Fig). Interestingly, while the HBx-specific antibody could only detect the FLAG-HBx cotransfected with pCMVHBVΔx in a 1:1 mass ratio (lane 6), co-transfection of FLAG-HBx at 10-fold and even 100-fold less amount also restored the transcription of pCMVHBVΔx to the similar or higher level as the wild type pCMVHBV (lanes 4–5). Furthermore, pCMVHBV per se produced undetectable HBx protein (lane 1). The above results indicate that the HBx-mediated activation of HBV plasmid transcription is very stoichiometrically efficient.

### Effect of HBx on transcription from cccDNA in HBV stably transfected cells

Previously, we have developed a tetracycline (tet)-inducible HBV stable cell line HepBHAe82 that produces pC mRNA and HA-tagged HBeAg protein in a cccDNA-dependent manner, allowing HA-HBeAg to serve as a surrogate marker for transcription activity of cccDNA [37]. In order to further investigate the role of HBx in HBV replication cycle in the context of cccDNA, we set out to establish a sibling inducible cell line producing HBV in the absence of HBx expression by following the similar strategy of HepBHAe82 cell line establishment as previously described [37]. To abrogate HBx expression from the HBV transgene, and cccDNA, if any, a premature stop codon-forming point mutation C1397T was introduced into the HBx ORF at amino acid position 8 without affecting the amino acid sequence of the overlapping pol gene (S2 Fig). Firstly, the wt and HBx-null HBV transgene plasmids, specifically pTREHBV-HAe and pTREHBVΔx-HAe, were individually transfected into HepG2 cells together with plasmid pTet-off expressing the tet-responsive transcriptional activator (tTA) to assess HBV replication competency. As shown in S3A Fig, both plasmids supported HBV RNA transcription and core DNA replication in transient transfection; moreover, consistent with the wt and HBx-null pCMVHBV plasmid transfection (S1 Fig), the HBx-null plasmid pTREHBVΔx-HAe produced a lower level of HBV RNA transcription and the subsequent DNA replication compared to pTREHBV-HAe, further confirming that HBx enhances plasmid-based transcription. Next, the HepG2 cells transfected by pTREHBVΔx-HAe and pTet-off were subjected to geneticin (G418) selection and colony expansion. A range of G418-resistant cell clones were selected and further tested for their tet-inducible HBV DNA replication, two cell clones with higher inducible levels of HBV replication were designated as HepBHAeΔx67 and HepBHAeΔx114, respectively, and prioritized to further assessment (S3B Fig). The presence of HBx-null mutation in HBV core DNA of both cell lines was confirmed by PCR sequencing.

**Fig 1:**
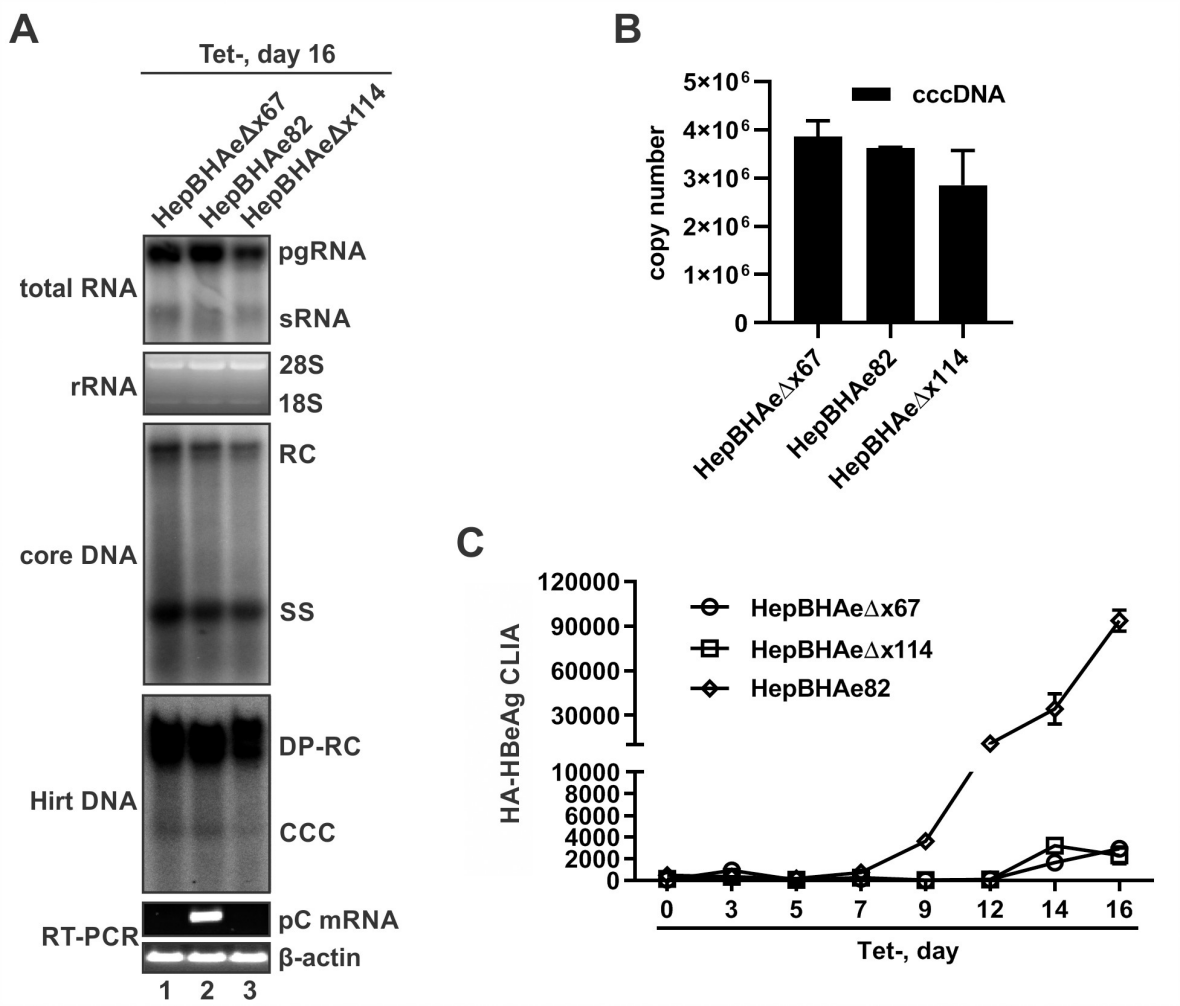
Comparative characterization of HBV replication, cccDNA formation and transcription, and HA-HBeAg production in HepBHAe82 cells and HBx-null HepBHAeΔx67 and HepBHAeΔx114 cell lines. The cell lines were seeded in 35mm-dish with density of 1.2×10^6^ cells/dish and induced for HBV replication in the absence of tet for 16 days, and subjected to the following analyses: (A) Panels from top to bottom: HBV total viral RNA, including pregenomic (pg) RNA and surface mRNA (sRNA), were detected by Northern blot. Cellular 28S and 18S ribosomal RNA (rRNA) serving as RNA loading control; HBV cytoplasmic core DNA and HBV Hirt DNA were detected by Southern blot. The relaxed circular (RC) DNA, single stranded (SS) DNA, deproteinated RC (DP-RC) DNA and cccDNA are indicated; HBV pCore (pC) mRNA was detected by reverse transcription (RT)-PCR gel electrophoresis, cellular β-actin mRNA RT-PCR served as loading control. (B) cccDNA extracted from each cell lines in a 35-mm dish were quantified for copy numbers by qPCR. (C) Supernatant HA-HBeAg produced by the three cell lines at indicated time points were measured by CLIA and plotted as a histogram (Mean ± SD, n = 3).

In the designed HBx-null HBV stable cell lines, HA-HBeAg is to be produced and released in a cccDNA-dependent manner (S2 Fig). To confirm that, the two selected clones HepBHAeΔx67 and HepBHAeΔx114 were compared with the HepBHAe82 for cccDNA formation, pC mRNA transcription, and HA-HBeAg expression. As shown in Fig 1, these three cell lines supported comparable levels of transgene-based pgRNA transcription regardless of the presence or absence of HBx expression (Fig 1A, top panel), which is consistent with previous studies showing that HBx stimulates gene expression selectively from extrachromosomal DNA templates [30,36]. Furthermore, though with slightly variable levels, HBV cytoplasmic core DNA and cccDNA were successfully detected in both wt and HBx-null cell lines, indicating that HBx is not indispensable for HBV DNA replication or cccDNA formation (Fig 1A, middle panels; and Fig 1B). In marked contrast, the levels of cccDNA-dependent pC mRNA and its translational product HA-HBeAg were significantly lower in HepBHAeΔx67 and HepBHAeΔx114 cells compared to HepBHAe82 cells (Fig 1A, lower panels; and Fig 1C), thus indicating an essential role of HBx in cccDNA transcription activity. In this regard, the slightly lower levels of total HBV RNA in HepBHAeΔx67 and HepBHAeΔx114 cells might be partly due to the loss of mRNA transcribed from cccDNA (Fig 1A, top panel), which, once formed, serves as a minor transcription template for HBV RNA transcription in addition to the major transgene template in HBV stable cell lines [38,39]. Considering that the viral productivity is higher in HepBHAeΔx67 cells compared to HepBHAeΔx114 cells (Fig 1), the former was chosen as an HBx loss-of-function model for further studies.

Next, we examined whether the HBx deficiency-associated phenotype in HepBHAeΔx67 cells could be reversible. To do that, HepBHAeΔx67 cells were stably transduced by a retroviral vector expressing HBx to generate the HepBHAeΔx67+HBx cell line for assessment of cccDNA transcription using pC mRNA RT-qPCR assay (S4A Fig). The result demonstrated that the pC mRNA transcription was rescued in HepBHAeΔx67+HBx cells, though still lower than that in the HepBHAe82 cells. Furthermore, the production of HA-HBeAg was also upregulated in HepBHAeΔx67+HBx cells compared to HepBHAeΔx67 (S4B Fig). Collectively, the results further suggested that HBx is required for cccDNA transcription in HBV stable cell lines.

### Epigenetic profiling of HBV cccDNA in the presence and absence of HBx

**Fig 2:**
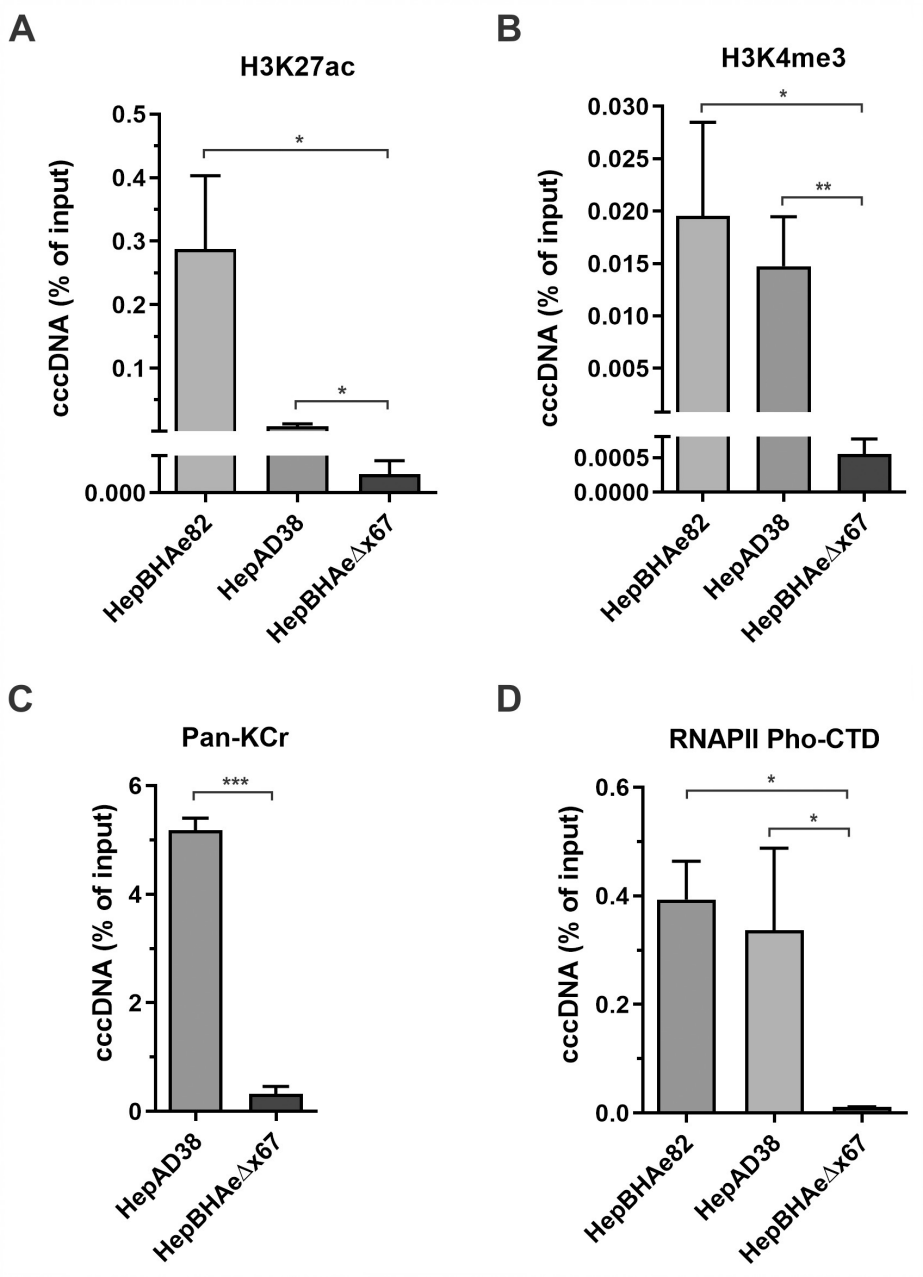
ChIP-qPCR analyses of HBV cccDNA-associated active epigenetic markers in the presence and absence of HBx. HBx-positive HBV cell line HepAD38 and HepBHAe82 and HBx-null HBV cell line HepBHAeΔx67 were induced in the absence of tet for 14 days. The association of (A) acetylated lysine 27 of histone H3 Lysine 27 (H3K27ac), (B) trimethylated lysine 4 of histone H3 (H3K4me3), (C) pan crotonylated lysine (Pan-Kcr), and (D) Phosphorylated C-terminal domain of RNA polymerase II (RNAPII pho-CTD) with cccDNA was analyzed by ChIP-qPCR and presented in percentage (%) of input (mean ± SEM, n = 3). *p<0.05, **p<0.01, ***p<0.001.

**Fig 3:**
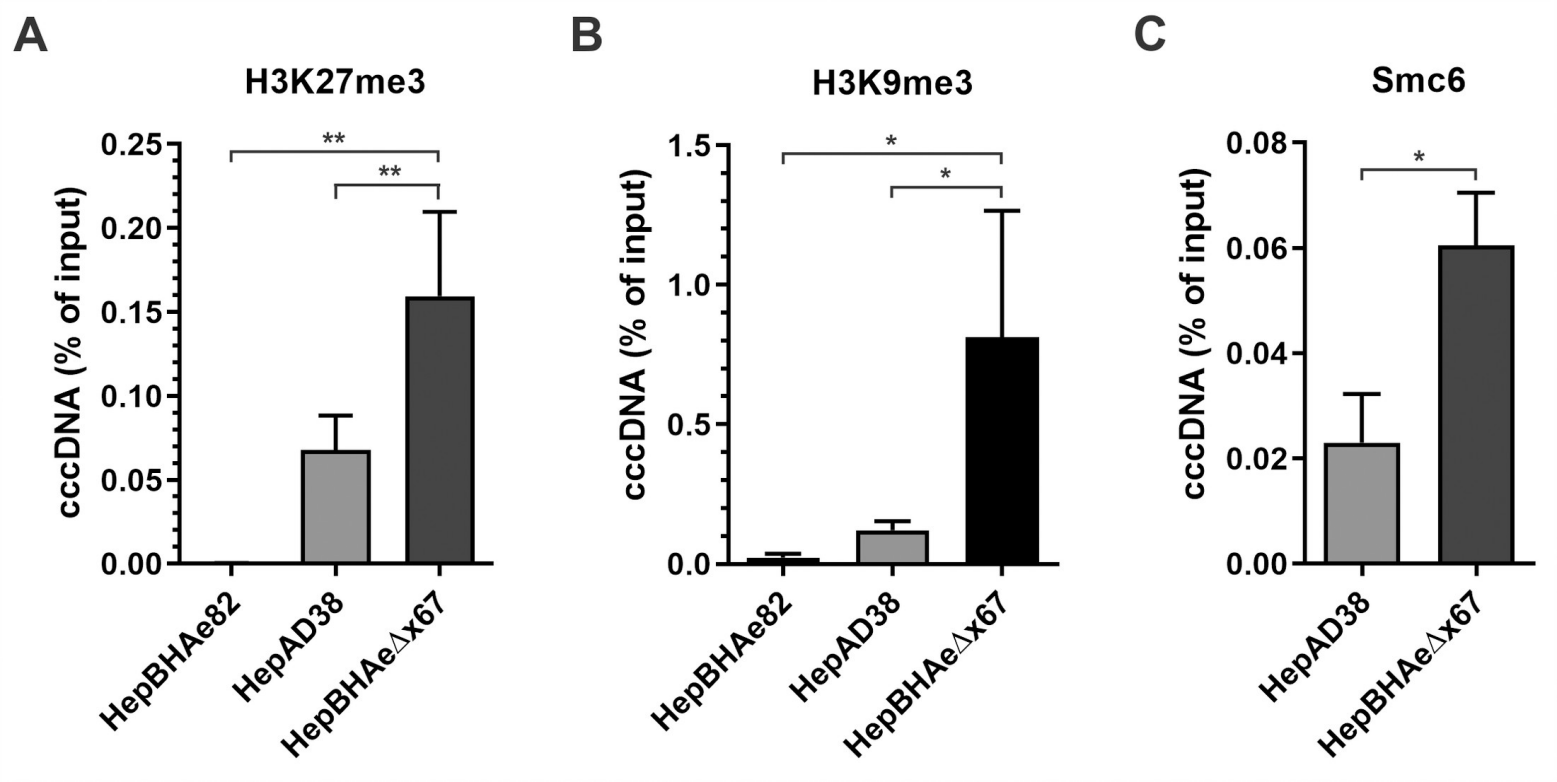
ChIP-qPCR analyses of HBV cccDNA-associated repressive markers/factors in the presence and absence of HBx. HBx-positive HBV cell line HepAD38 and HepBHAe82 and HBx-null HBV cell line HepBHAeΔx67 were induced in the absence of tet for 14 days. The association of cccDNA with (A) trimethylated lysine 27 of histone H3 (H3K27me3), a repressive histone PTM, (B) trimethylated lysine 9 of histone H3 (H3K9me3), a repressive histone PTM, and (C) Smc6, a reported host restriction factor of HBV cccDNA, was analyzed by ChIP-qPCR and presented in percentage (%) of input (mean ± SEM, n = 3). *p<0.05, **p<0.01.

HBV cccDNA episome possesses chromatin structure, harboring hijacked host histone- and non-histone proteins, and undergoing epigenetic regulations through histone posttranslational modifications (PTMs) [9,18]. To characterize the epigenetic profile of cccDNA minichromosomes produced in the inducible cell lines with and without HBx protein, chromatin immunoprecipitation coupled with quantitative PCR (ChIP-qPCR) method has been applied. Occupancy of activating histone PTMs, such as Histone H3 Lysine 27 acetylation (H3K27ac), Histone H3 Lysine 4 three-methylation (H3K4me3), and pan-lysine crotonylation (Pan-Kcr), was drastically decreased on cccDNA in HepBHAeΔx67 cells in absence of HBx, compared to that in HepBHAe82 cells as well as another HBx-positive HBV stable cell line HepAD38 (Fig 2A–2C). Along with that, the active form of RNA polymerase II with phosphorylated C-terminal domain (RNAPII pho-CTD) was highly enriched on cccDNA in the presence of HBx, demonstrating an active transcription state of cccDNA (Fig 2D). Upon comparison of the two chromatin repressive markers, Histone H3 Lysine 27 three-methylated (H3K27me3) and Histone H3 Lysine 9 three-methylated (H3K9me3) on cccDNA from HepBHAeΔx67 versus wt HBV-producing cell lines, we confirmed the higher enrichment of the repressive PTMs, along with a host restriction factor of HBV cccDNA, Smc6 [30,31], on the HepBHAeΔx67-derived cccDNA (Fig 3). The active H3K27ac and repressive H3K27me3 histone PTMs comparative profiling was then extended to other two HepBHAe82 sibling clones, specifically HepBHAe1 and HepBHAe45 [37], which confirmed that the phenotype of cccDNA-bound histones in HepBHAe82 cell line is not cell clone-specific (S5 Fig). Profiling of the other heterochromatin markers and repressive chromatin modification “writers”, such as the Histone H3 Lysine 9 di-methylated (H3K9me2), a linker histone H1, SET Domain Bifurcated Histone Lysine Methyltransferase 1 (SETDB1), and SETDB2, were demonstrated to be highly enriched on the HBx-deficient cccDNA (S6 Fig).

**Fig 4:**
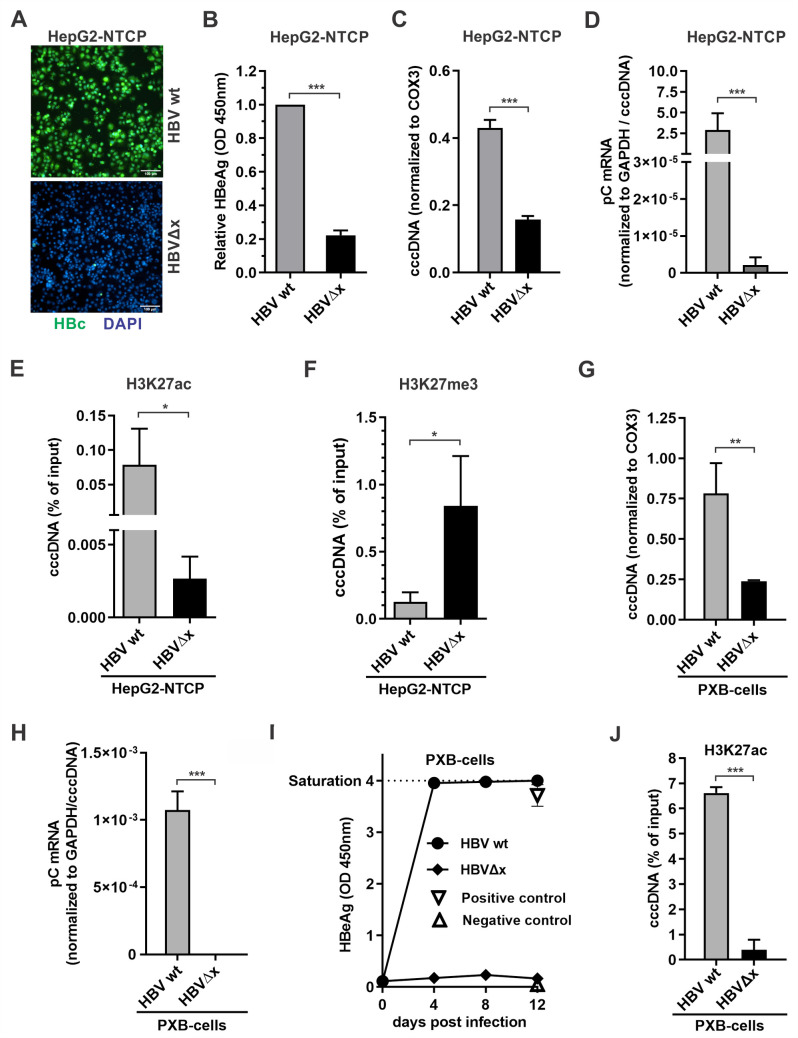
Characterization of the dependency of cccDNA transcription on HBx during HBV infection. (A-F) HepG2-NTCP cells were infected with wt HBV and HBVΔx viral particles at 500 vge/cell for 10 days, and the following assays were performed: A) Intracellular HBV core protein (HBc) was detected by immunofluorescence, cell nuclei were stained by DAPI. Scale bar = 100 μm; B) Supernatant HBeAg was measured by ELISA (OD 450nm values; mean ± SD, n = 3); C) Levels of cccDNA were detected by qPCR and normalized to COX3 (mean ± SEM, n = 3); D) The relative levels of intracellular HBV pC mRNA was detected by qPCR and normalized to GAPDH and cccDNA (mean ± SEM, n = 3); E) The cccDNA-associated histone PTM H3K27ac was assayed by ChIP-qPCR and shown in percentage (%) of input (**Δ**Cq; mean ± SEM, n = 3); F) The cccDNA-associated histone PTM H3K27me3 was analyzed by ChIP-qPCR and shown in percentage (%) of input (mean ± SEM, n = 3). (G-J) PXB-cells cells were infected with HBV wt and HBVΔx viral particles at 500 vge/cell for 12 days. G) Levels of cccDNA were detected by qPCR and normalized to COX3 (mean ± SEM, n = 3); H) HBV pC mRNA and I) HBeAg were analyzed as described above; J) cccDNA-associated H3K27ac was analyzed by ChIP-qPCR and shown in percentage (%) of input (mean ± SEM, n = 3). *p<0.05, **p<0.01, ***p<0.001.

We then further assessed the HBx-dependent cccDNA epigenetic profiling in HBV cell infection models, including HepG2-NTCP cells and primary human hepatocyte (PHH)-derived PXB-cells. As shown in Fig 4, upon inoculation of wt HBV and HBx-null HBVΔx in HepG2-NTCP cells, the HBVΔx virus exhibited significantly lower infectivity as evidenced by intracellular HBV core protein (HBc) immunofluorescence and supernatant HBeAg ELISA (panels A and B, respectively). While the HBVΔx virus infection produced approximately 3-fold less cccDNA compared to wt HBV (panel C), when the relative levels of pC mRNA were quantified and normalized to cccDNA levels, we found that the transcriptional activity of HBVΔx-derived cccDNA was remarkably low (panel D). The above phenotypes of HBVΔx virus cccDNA production and transcription are consistent with a previous study [19]. Interestingly, the silenced viral transcription in HBVΔx-infected HepG2-NTCP cells was correlated with reduced H3K27ac and enriched H3K27me3 occupancy on cccDNA (panels E and F). Furthermore, the HBVΔx virus was also found to be epigenetically silenced in PXB-cells through analyzing pC mRNA, HBeAg, and cccDNA-associated H3K27ac in comparison with wt HBV infection (panels G-J).

### Mass spectrometry identification of potential cccDNA-bound proteins in the presence and absence of HBx

The epigenetic profiling of HBV-producing cell lines and HBV-infected cells has demonstrated a crucial role of HBx protein in maintenance of active cccDNA minichromosome euchromatin (Figs 2–4, S5 and S6). To identify potential host factors participating in cccDNA episome regulation, we designed the following pulldown of cccDNA-associated proteins.

It has been reported that HBc protein is a component of cccDNA minichromosome through direct DNA binding and promotes a permissive epigenetic state of cccDNA [40], however, growing evidence has revealed its dispensable role in cccDNA transcription, at least the *de novo* HBc expression [41–43]. Therefore, we used anti-HBc antibodies to obtain potential cccDNA-proteins complex precipitate on the surface of paramagnetic beads. The pulldown from nuclear fraction of induced HepAD38 and HepBHAeΔx67 was followed by on-beads digestion and liquid chromatography with tandem mass spectrometry (LC-MS/MS) protein sequencing. NCBI human database was used to identify the sequenced peptides. Beads-only variables were subtracted as background control for each cell line (S7A Fig).

The comparative proteomic profiling of the wt and HBx-null HBV cell lines has identified a list of non-histone hits in three groups: (1) sharing specificity in both phenotypes (HBc-interacting proteins and/or cccDNA scaffold proteins); (2) specific for cells containing transcription-active HBV cccDNA (potential cccDNA enhancers); (3) and specific for cells containing transcription-inactive cccDNA in the absence of HBx protein (potential cccDNA silencers) (S7B Fig and S1 Table). One of the HepBHAeΔx67-specific hits, which drew our closer attention, was the high mobility group box 1 protein (HMGB1), a nuclear non-histone DNA-binding protein that regulates nucleosome dynamics and chromosomal stability, gene transcription and V(D)J recombination, DNA repair and telomere maintenance [44,45].

### HMGB1 is enriched on cccDNA in the absence of HBx

**Fig 5:**
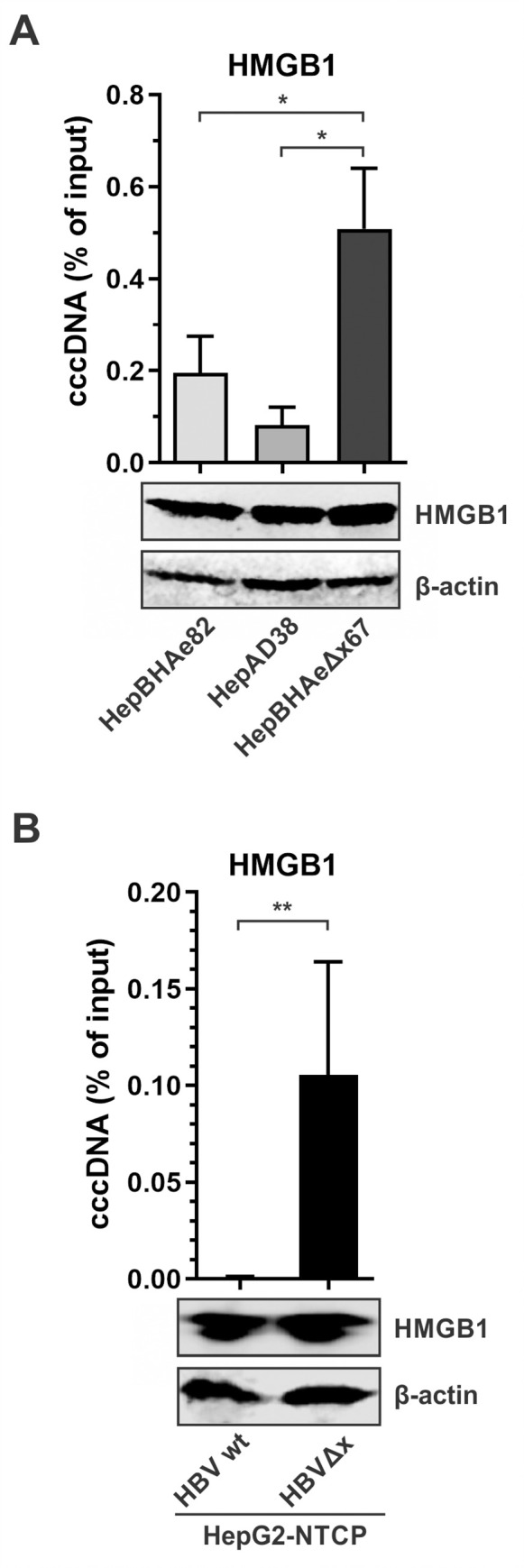
HMGB1 is highly enriched on cccDNA in the absence of HBx. (A) HepAD38, HepBHAe82, and HepBHAeΔx67 cells were induced for HBV production for 14 days. The expression of endogenous HMGB1 was detected by Western blot, β-actin served as a loading control. The association of HMGB1 with cccDNA was analyzed by ChIP-qPCR and presented in percentage (%) of input (mean ± SEM, n = 3). (B) HepG2-NTCP cells were infected with wt HBV or HBV**Δ**x for 10 days and subjected to the same analyses as aforementioned in (A). *p<0.05, **p<0.01.

To validate the pulldown-mass spectrometry data, a ChIP-qPCR for HMGB1 occupancy was performed on HBx-expressing HepBHAe82 and HepAD38 cell lines, and HBx-null HepBHAeΔx67 cells, as well as HepG2-NTCP cells infected with wt HBV and HBVΔx, respectively. The results demonstrated that, indeed, HMGB1 was highly enriched on cccDNA minichromosome in the absence of HBx expression without total cellular HMGB1 abundancy fluctuation (Fig 5). Furthermore, the level of cccDNA-associated HMGB1 was significantly reduced in HepBHAeΔx67+HBx cells (S8 Fig).

### HBx binds to HMGB1 and partially alters its subcellular localization

**Fig 6:**
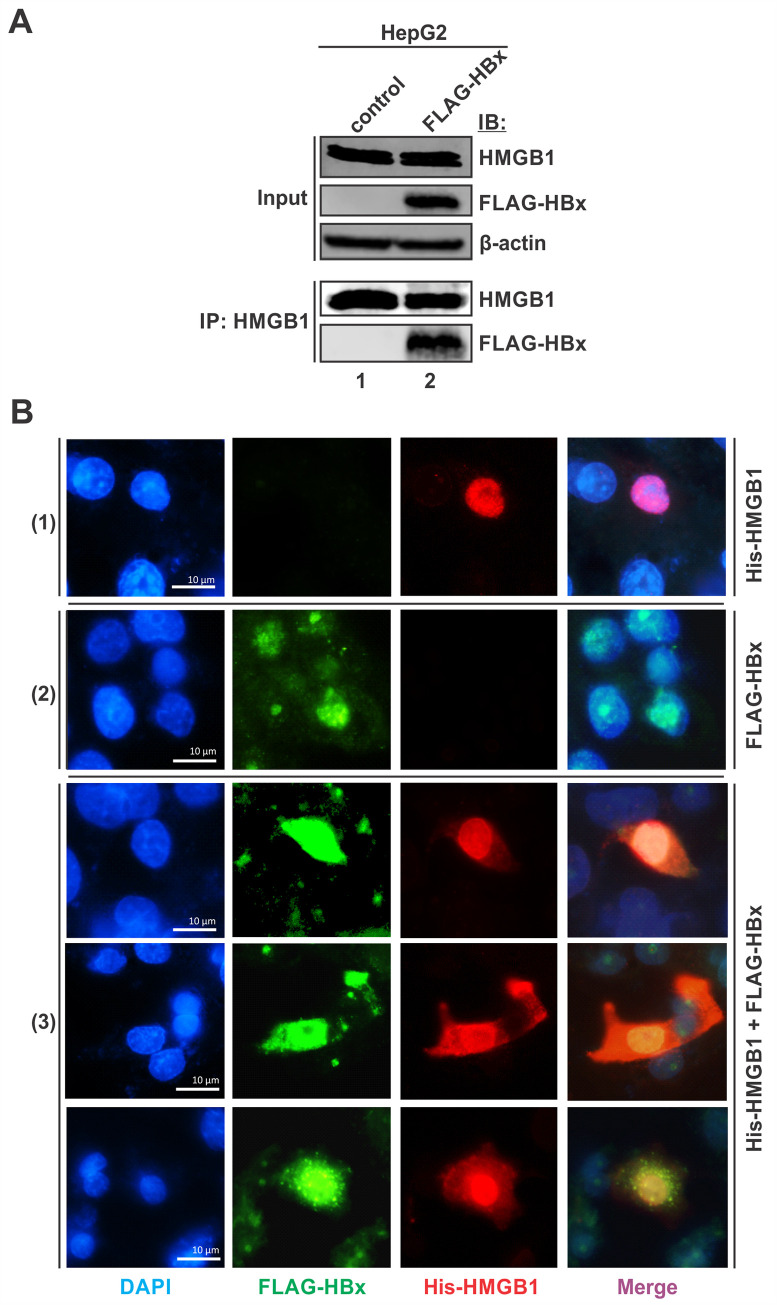
HMGB1 and HBx interact in cell culture and alter the protein subcellular localization. (A) HepG2 cells were transfected with control vector or FLAG-HBx for 3 days. The expression of endogenous HMGB1 and transfected FLAG-HBx were detected by Western blot, β-actin served as a loading control. Co-immunoprecipitation of endogenous HMGB1 was performed, and the immunoprecipitated HMGB1 and FLAG-HBx were detected by Western blot. (B) Cells were transfected with (1) His-HMGB1 or (2) FLAG-HBx or (3) both for 3 days, followed by immunofluorescence microscopy analyses of His-HMGB1 (stained in red) and FLAG-HBx (stained in green), their colocalization was shown as bright yellow to orange signals. Cell nuclei were stained by DAPI (blue). Scale bar = 10 μm.

The enrichment of HMGB1 on cccDNA in the absence of HBx indicated a possible interplay between these two proteins. To assess the potential interaction of viral HBx and host HMGB1 proteins, co-immunoprecipitation and immunofluorescence assays were performed. As shown in Fig 6A, the immunoprecipitation of endogenous HMGB1 co-precipitated down the overexpressed FLAG-HBx. The ectopically expressed His-HMGB1 exhibited a predominant nuclear localization in the absence of HBx (Fig 6B, panel 1), which is consistent with the reported nuclear localization of HMGB1 under normal conditions [44]. While the overexpressed FLAG-HBx was localized in both the nucleus (major) and cytoplasm (minor) (Fig 6B, panel 2), it colocalized with the co-expressed His-HMGB1 mainly in nucleus, and a portion of His-HMGB1 was detected in cytoplasm (Fig 6B, panel 3). Thus, the results indicate that HBx interacts with nuclear HMGB1 and induces a partial nucleocytoplasmic translocation of HMGB1, preventing the interaction between HMGB1 and cccDNA to enable a permissive epigenetic state of cccDNA.

### HMGB1 inhibition reactivates cccDNA transcription in the absence of HBx

To functionally validate the role of HMGB1 in silencing cccDNA, firstly, HMGB1 was pharmacologically inhibited in HepBHAeΔx67 cells with glycyrrhizin, a natural compound that inhibits HMGB1 through directly binding to the concave surface of HMGB1 protein [46]. As shown in S9 Fig, glycyrrhizin treatment resulted in a slight upregulation of the cccDNA-dependent pC mRNA transcription in HepBHAeΔx67 cells, indicating that inhibiting HMGB1 could alleviate the silencing effect of HBx-loss on cccDNA. It is worth noting that glycyrrhizin has been used to treat chronic hepatitis B in Japan, the therapeutic effect of glycyrrhizin may be related to the suppression of HBsAg sialylation and secretion as well as the drug’s anti-inflammatory activity [47,48]. Nonetheless, the observed pro-HBV activity of glycyrrhizin in this study, albeit weak, may be mechanistically distinct from its previously reported anti-HBV effects.

**Fig 7:**
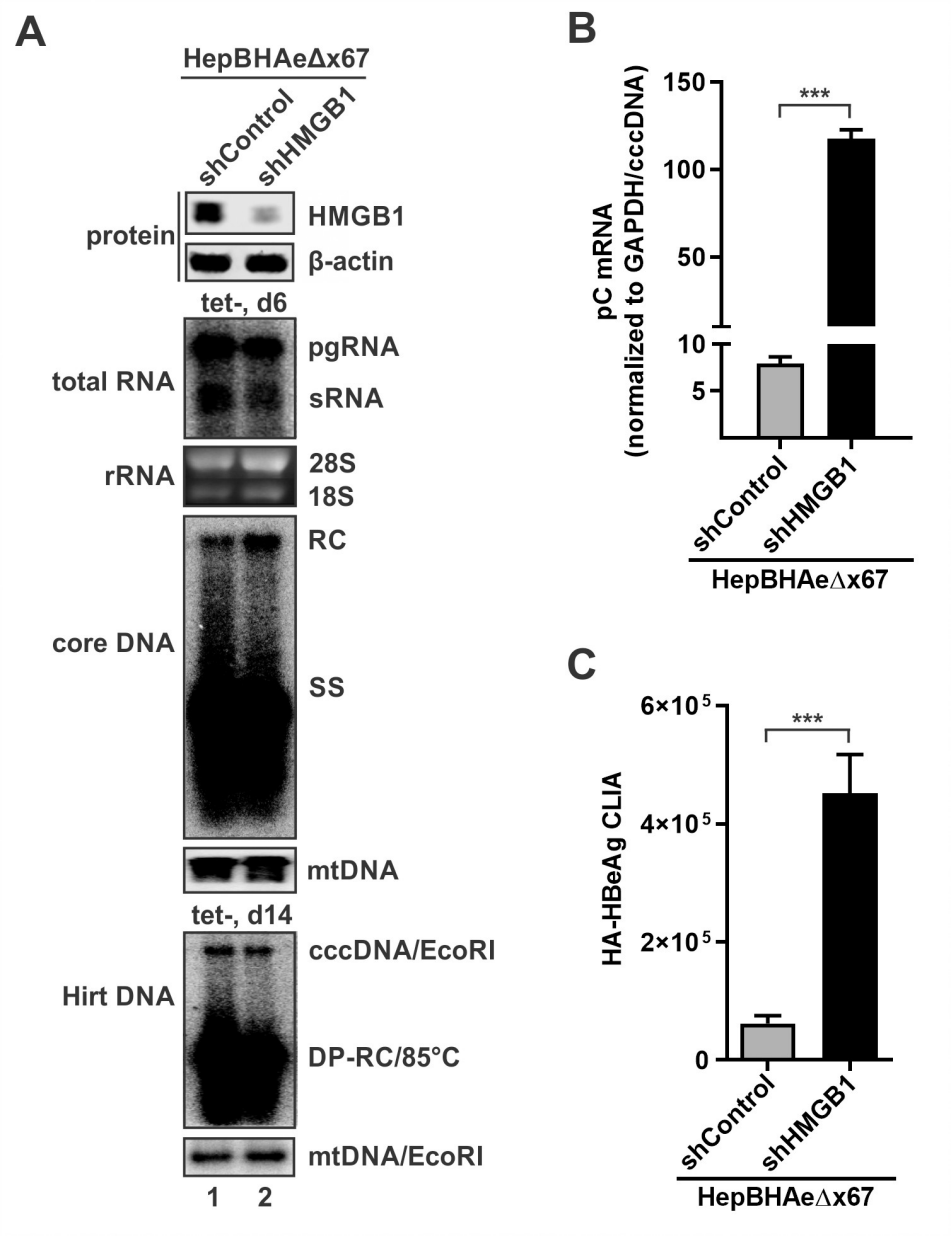
HMGB1 knockdown rescues cccDNA transcriptional activity in the absence of HBx. (A) Assessment of the HepBHAeΔx67-shControl and HepBHAeΔx67-shHMGB1 cell lines. The knockdown of HMGB1 in HepBHAeΔx67-shHMGB1 cells was validated by Western blot, β-actin served as loading control. After 6-days induction, HBV total RNA was analyzed by Northern blot, cellular 28S and 18S rRNA served as loading control; HBV cytoplasmic core DNA was analyzed by Southern blot, mitochondrial (mt) DNA served as a loading control. After 14-days induction, total cellular Hirt DNA was heat-denatured, followed by EcoRI digestion, and then subjected to Southern blot analyses of HBV deproteinated rcDNA (DP-RC), linearized cccDNA, and mtDNA. (B) The relative levels of HBV cccDNA-dependent pC mRNA in HepBHAeΔx67-shControl and HepBHAeΔx67-shHMGB1 cells were analyzed by qPCR and normalized to cellular GAPDH and cccDNA (mean ± SEM, n = 3). (C) Supernatant HA-HBeAg was detected by CLIA (mean ± SEM, n = 3). ***p<0.001.

**Fig 8:**
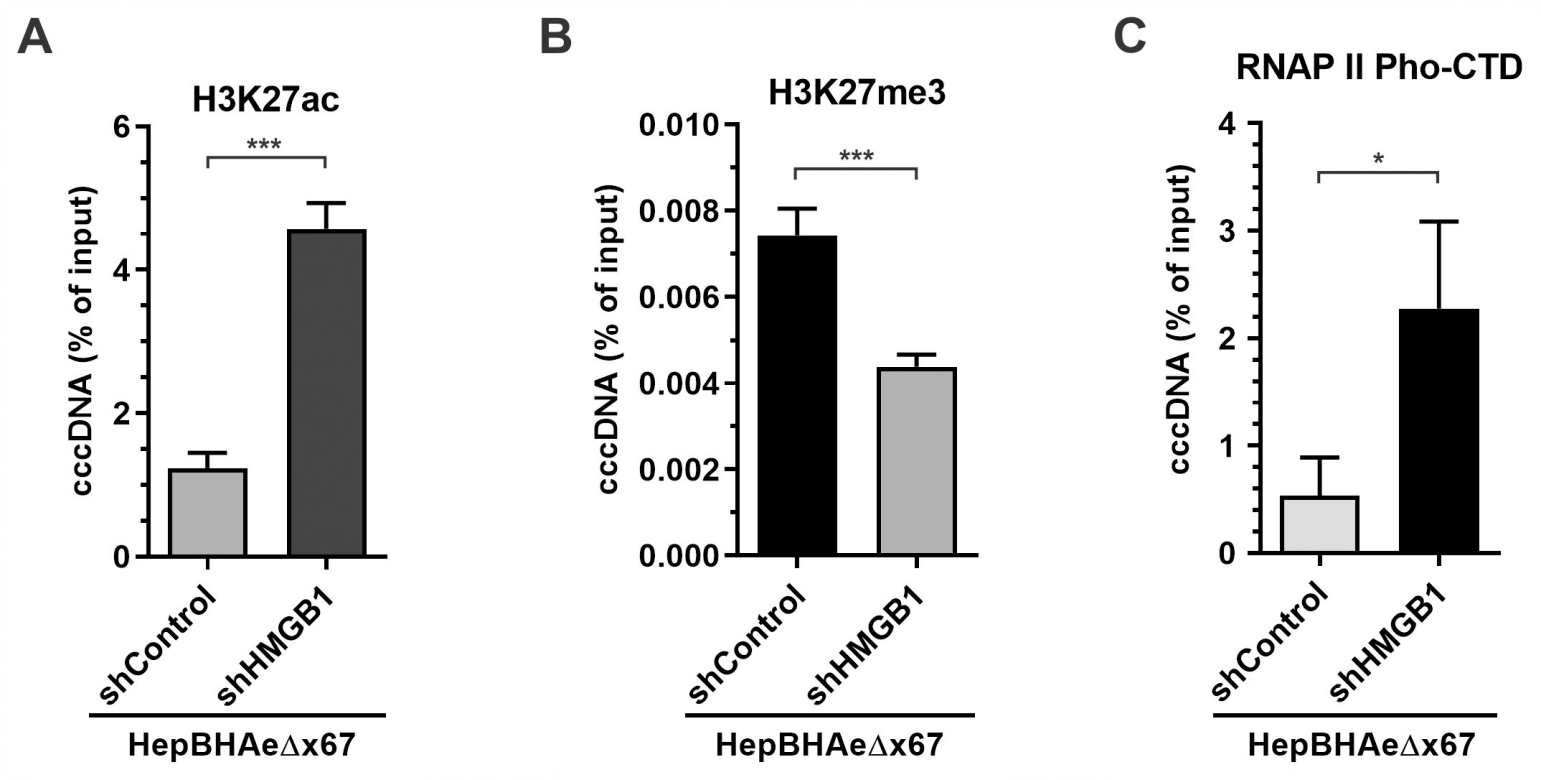
Effect of HMGB1 knockdown on cccDNA-associated epigenetic markers in the absence of HBx. HepBHAeΔx67-shControl and HepBHAeΔx67-shHMGB1 cells were cultured in tet-free medium to induce HBV replication for 14 days, the association of (A) H3K27ac, (B) H3K27me3, and (C) RNAPII pho-CTD with cccDNA was analyzed by ChIP-qPCR and plotted in percentage (%) of input (mean ± SEM, n = 3). *p<0.05, ***p<0.001.

To further downregulate HMGB1, lenti-shRNA stable knock-down was performed in HepBHAeΔx67 cells to generate HepBHAeΔx67-shControl and HepBHAeΔx67-shHMGB1 cell lines. The HBV transgene-derived productivities of viral RNA, core DNA, and cccDNA were comparable in both cell lines, indicating that HMGB1 does not significantly affect the transcription of integrated HBV or the biosynthesis of HBV core DNA and cccDNA (Fig 7A). However, the cccDNA-dependent pC mRNA and HA-HBeAg were significantly upregulated in HepBHAeΔx67-shHMGB1 cells compared to HepBHAeΔx67-shControl cells, suggesting a re-activation of cccDNA transcription upon HMGB1 inhibition (Fig 7B and 7C). Furthermore, epigenetic profiling of cccDNA derived from the HepBHAeΔx67-shControl and HepBHAeΔx67-shHMGB1 cells demonstrated acquisition of the active histone PTM H3K27ac, loss of the repressive H3K27me3 upon downregulation of HMGB1 and recruitment of RNAPII pho-CTD (Fig 8A–8C). Overall, HMGB1 downregulation in the absence of HBx protein led to transcriptional reactivation of cccDNA, including switching of the viral minichromosome into the active chromatin mode with active histone PTMs and recruitment of the active RNA transcription machinery.

### HMGB1 inhibition epigenetically upregulates cccDNA transcription in HBV infection

**Fig 9:**
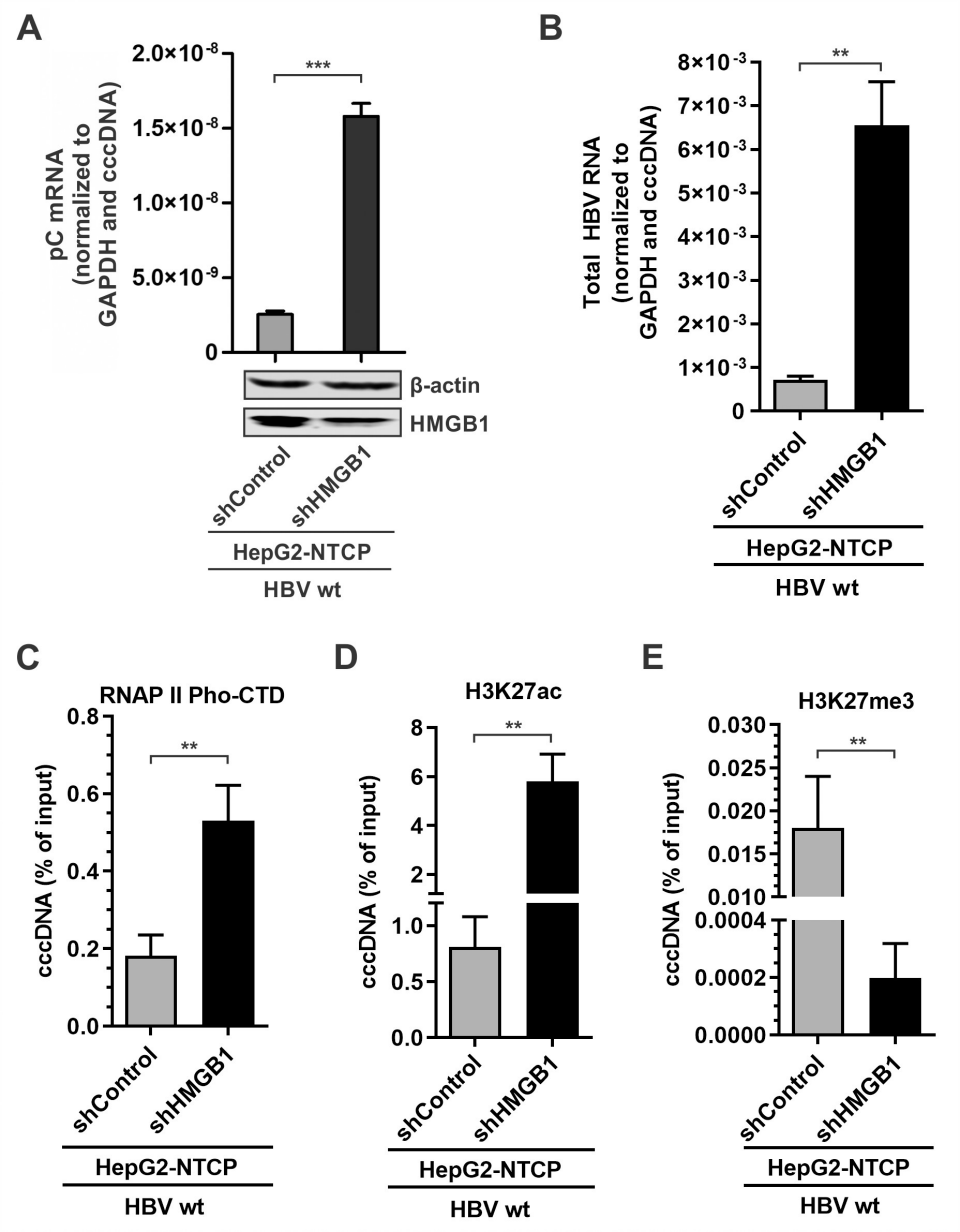
Effect of HMGB1 knockdown on cccDNA transcription during wt HBV infection. HepG2-NTCP-shControl and HepG2-NTCP-shHMGB1 cells were infected by wt HBV at 500 vge/cell for 10 days. (A-B) The levels of HMGB1 protein expression were analyzed by Western blot. HBV pC mRNA and total HBV RNA were analyzed by qPCR and normalized to GAPDH mRNA and HBV cccDNA (mean ± SEM, n = 3). The enrichment of RNAPII pho-CTD (C), H3K27ac (D), and H3K27me3 (E) on cccDNA was analyzed by ChIP-qPCR as presented in percentage (%) of input (mean ± SEM, n = 3). **p<0.01, ***p<0.001.

**Fig 10:**
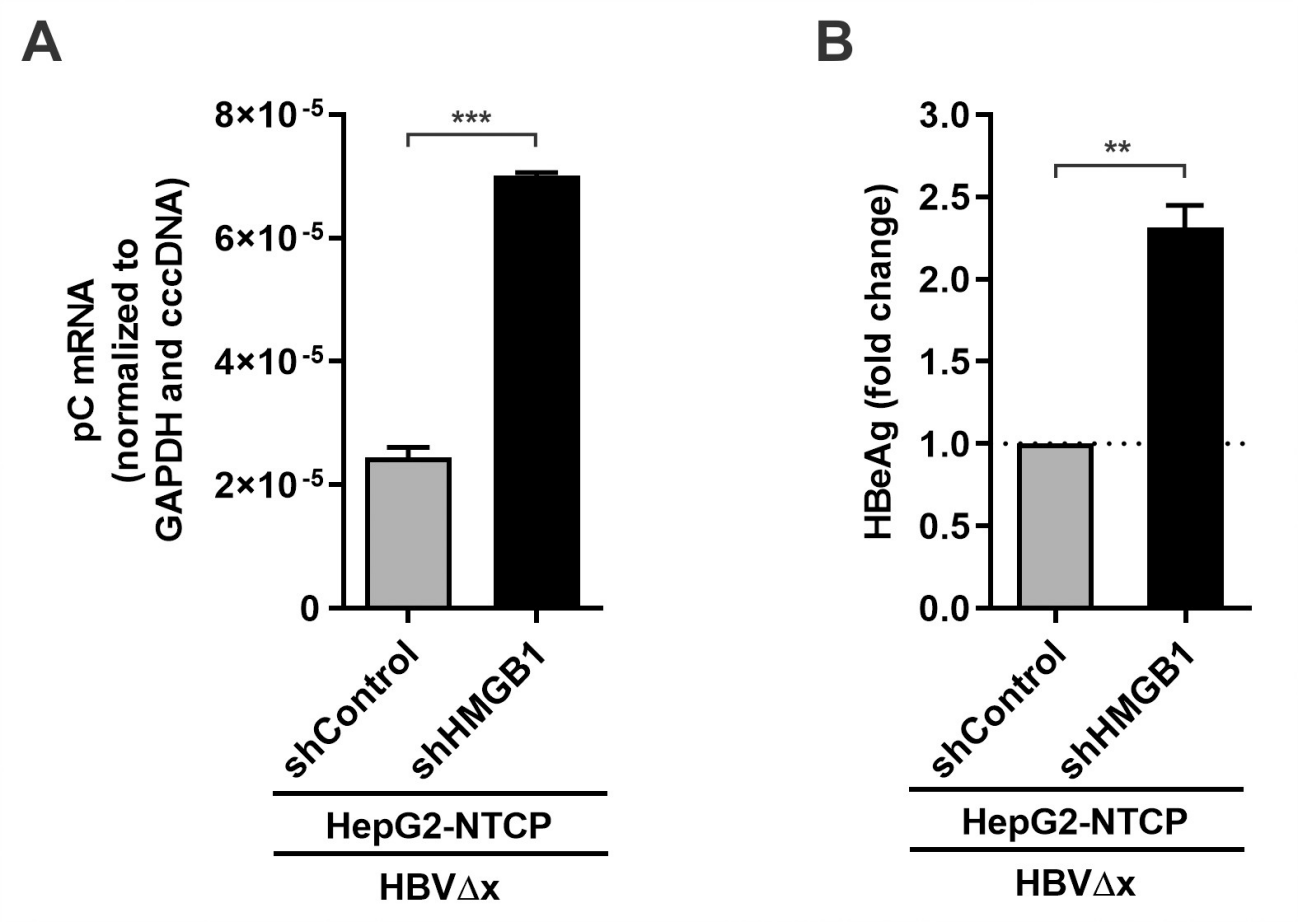
HMGB1 knockdown restores HBV gene expression in HBVΔx-infected cells. HepG2-NTCP-shControl and HepG2-NTCP-shHMGB1 cells were infected by HBVΔx at 500 vge/cell for 6 days. (A) Intracellular HBV pC mRNA was quantified by qPCR and normalized to GAPDH mRNA and HBV cccDNA (mean ± SEM, n = 3); (B) The levels of HBeAg in supernatant were detected by ELISA and expressed as fold changes (mean ± SEM, n = 3). **p<0.01, ***p<0.001.

To further validate the restriction effect of HMGB1 on cccDNA transcription in the viral infection model, the HepG2-NTCP-shControl and HepG2-NTCP-shHMGB1 cell lines were established. The cells were infected with HBV, the pC mRNA and total HBV RNA qPCR assay confirmed the elevated transcriptional activity of the HBV cccDNA upon HMGB1 knock-down (Fig 9A and 9B). ChIP-qPCR assay detected elevated active RNAPII and H3K27ac (Fig 9C and 9D**)** but decreased H3K27me3 (Fig 9E**)** enrichment on cccDNA under mitigated HMGB1 expression. Furthermore, knockdown of HMGB1 upregulated pC mRNA transcription and subsequent HBeAg production in HBVΔx-infected HepG2-NTCP cells (Fig 10). These results therefore suggest that HMGB1 is an intrinsic host restriction factor of HBV infection through epigenetically inhibiting cccDNA transcription.

## Discussion

The cccDNA episome, a *bona fide* source of HBV infection persistence, is transcriptionally regulated by the virally encoded HBx protein. HBx is a small 154-aa long protein of ~17 kDa, known to be indispensable for cccDNA transcription initiation and maintenance during HBV infection *in vitro* and in human hepatocyte chimeric mice models [17,19,49]. Interestingly, HBx can also transactivate gene expression from extrachromosomal DNA templates in general, such as transfected plasmid DNA, regardless of HBV or non-HBV promoters [36]. In this study, we have systematically validated the transactivator role of HBx in HBV transient and stable transfection systems (Figs S1 and 1), as well as in HBV-infected HepG2-NTCP cells and PHH-derived PXB-cells (Fig 4). Among them, the wt HBV stable cell line, HepBHAe82, and the newly established HBx-null HBV stable cell line, HepBHAeΔx67, support the production of HA-HBeAg in a cccDNA- and HBx-dependent manner, which could serve as a convenient and powerful cell system for studying the interplay between HBx and cccDNA.

Accumulating evidence has suggested that HBx transactivates the cccDNA minichromosome via epigenetic mechanisms. It has been demonstrated that HBx recruits histone acetyltransferases (HATs) (p300, CBP, GCN5/PCAF) to cccDNA, prevents binding of histone deacetylases (HDACs) (HDAC1, SIRT1, SIRT3) to viral promotors and pro-viral host genes, and inhibits repressive PTMs writers (SETDB1, PRMT1), by which coordinately shape an epigenetically permissive landscape of cccDNA minichromosome for transcription [18,24,25,50]. Through comparative profiling of the major histone PTMs associated with wt and HBx-null cccDNA in HBV stable cell lines and infected cells, we demonstrated that HBx is responsible for the enrichment of active histone PTMs on cccDNA as well the depletion of repressive histone PTMs (Figs 3, 4 and S6). Consistent with the previous ChIP-seq studies, the enrichment of repressive histone PTMs on cccDNA is not as prominent as the active ones, suggesting presumable epigenetic regulation of cccDNA minichromosome *via* active histone PTMs [51,52]. On the other hand, the stoichiometry and dynamics of histone PTMs on cccDNA so far have not been characterized quantitatively. Taking this into consideration, it is possible that even minor heterochromatinization through posing repressive histone PTMs together with a lack of activating histone PTMs might drastically reduce chromatin accessibility to transcriptional machinery.

As an epigenetic factor, HBx protein, even at low abundance inside the infected hepatocytes, possesses a high potential and wide network of interaction with host proteins as well as access to viral and host regulatory DNA regions [20,23,53–56]. In the present study, we, for the first time, compared the possible cccDNA-associated proteomes in the presence and absence of HBx, and identified a number of protein “hits” enriched on the transcriptionally repressed HBx-null cccDNA, which are potential cccDNA silencers antagonized by HBx (S7B Fig and S1 Table). We prioritized one hit, namely HMGB1, for further study due to its reported DNA binding activity and involvement in chromatin remodeling and transcription regulation [44].

HMGB1 is a small 215 aa cellular protein of ~25 kDa belonging to the highly conserved high mobility group (HMG) proteins superfamily [57]. HMGB1 has a tripartite structure composed of two homologous positively charged DNA-binding domains (the HMG boxes A and B), and a highly acidic carboxyl terminus of aspartic and glutamic acids. The ChIP-qPCR assay demonstrated an association of HMGB1 with cccDNA in the absence of HBx (Fig 5), perhaps due to its DNA binding property. However, there is no clearly established DNA sequence motif for HMGB1’s DNA binding specificity, but it has been shown that HMGB1 prefers pre-bent DNA for binding or such non-trivial structures as four-way junctions or DNA minicircles [58,59]. The sensing of minicircle DNA by HMGB1 is consistent with its interaction with HBV cccDNA, which is a circular DNA molecule. Nonetheless, whether there is a specific HBV DNA sequence conferring the binding of HMGB1 to cccDNA remains to be investigated, it is also possible that HMGB1 is recruited to cccDNA *via* other DNA binding protein(s).

HMGB1 protein has three general hypostases corresponding to its localization: (1) in the extracellular environment (PAMPs/MAMPs and DAMPs signaling, multiple receptors ligand, pro-inflammatory cytokine, stress response, autophagy and apoptosis regulator, DAMP clinical marker and anti-cancer target); (2) in cytoplasm or mitochondria (increases autophagy, inhibits apoptosis, regulates mitochondrial morphology and function); (3) and in the nucleus (DNA chaperone, nucleosome dynamics and chromosomal stability; gene transcription and V(D)J recombination; DNA repair and telomere maintenance) (see reviews [44,45,60]). Here, we focused on HMGB1 activities in the intranuclear compartment, where it colocalizes with and targets cccDNA. Considering that HMGB1 is enriched on cccDNA in the absence of HBx, we hypothesized that HBx prevents the interaction between HMGB1 and cccDNA. In line with this hypothesis, HBx was found to be interacting with HMGB1 through both co-immunoprecipitation assay and microscopic colocalization (Fig 6). Interestingly, while both HBx and HMGB1 are predominately localized in nucleus when expressed alone, HBx expression could partially induce a nuclear-cytoplasmic translocation of HMGB1, indicating that HBx may inhibit HMGB1-cccDNA interaction by directly binding to HMGB1 and/or blocking the nuclear localization of HMGB1. In partial agreement with these observations, a previous study demonstrated that the overexpressed HBx upregulates HMGB1 production and promotes its hyperacetylation by HDAC1 inside the nuclei, leading to a significant translocation of HMGB1 to the cytoplasm. Then, cytoplasmic HBx meets and binds HMGB1 to triggers autophagy in hepatocytes [61]. However, we did not observe obvious change of the steady state level of HMGB1 upon wt HBV replication/infection or HBx overexpression, and the HBx-mediated HMGB1 translocation was rather a minor effect (Figs 5 and 6), thus, the detailed mechanism underlying HBx-mediated blockage of HMGB1-cccDNA interaction awaits further investigation.

To assess the potential inhibitory effect of HMGB1 on cccDNA, we downregulated HMGB1 in HBx-deficient cell line HepBHAe67Δx by shRNA and demonstrated the heterochromatinized cccDNA underwent transcription recovery (Fig 7). The reactivation of cccDNA was characterized with cccDNA episome euchromatinization, with corresponding removal of repressive histone PTMs and switching to transcription activating histone PTM profiles (Fig 8). The results suggested a counteractive nature of HBx and HMGB1 inter-relationship, and the latter as an epigenetic silencer of HBV cccDNA. It also demonstrated that cccDNA may be effectively transcribed even in the absence of usually indispensable HBx upon having the intrinsic anti-viral mechanisms inactivated or inhibited to some extent. We then further explored the effect of HMGB1 on cccDNA transcription in HBV infection setting. HBV infection of HepG2-NTCP-shControl and HepG2-NTCP-shHMGB1 cells has demonstrated that, in the cells with abrogated HMGB1 expression, cccDNA transcription was elevated and its epigenetic profile acquired even stronger permissive pattern: with higher enrichment of RNAP II Pho-CTD and H3K27ac, and further reduction of H3K27me3 (Figs 9 and 10). Taken together, these data suggest that HMGB1 is an intrinsic host restriction factor for cccDNA transcription *via* epigenetic silencing mechanisms, which can be counteracted by HBx.

Interestingly, HepG2-NTCP-shHMGB1 clone selection has demonstrated an inverse relationship between HMGB1 knock-down extent and the cell viability and the cell division cycle activity, *i*.*e*. the more efficient knock-down was, the less viable clones became. This can explain our unsuccessful HMGB1 knock-out in HepG2-NTCP cells, which goes in line with lethality of HMGB1 deficiency shown in newborn mice [62], and suggests HMGB1 essentiality as a house-keeping gene.

HMGB1 is well-known for its extracellular activity as a DAMP alarmin, functioning in intercellular milieu. However, no strong paradigm of its intracellular role during viral infections has been established, and previous studies demonstrated that HMGB1 can serve as either antagonist or protagonist for viruses. For instance, during adenoviral infection, HMGB1 mediates antiviral immune response, and is affected by the viral histone-like viral protein VII [63]; on the other hand, for the gamma-2 herpesviruses, such as Kaposi’s sarcoma-associated herpesvirus (KSHV) and murine gamma herpesvirus 68 (MHV-68), HMGB1 supports the infection by activation of the viral immediate-early replication and transcription activator (RTA) [64]. In HIV-1 and HCV infections, it has been reported that HMGB1 exerted proviral or antiviral function depending on cell types, steps of viral lifecycle, and experimental models [65–68]. The proviral HMGB1 activity has also been shown through DNA transposition enhancement for systems like herpes simplex virus/sleeping beauty amplicons [69]. Recently, HMGB1 was identified as a proviral factor for SARS-CoV-2 infection through regulating viral receptor ACE2 expression [70]. In our study, the HMGB1-mediated epigenetic regulation of HBV cccDNA transcription represents a novel antiviral activity of HMGB1. Whether HMGB1 plays a similar role in regulating other DNA virus possessing episomal circular DNA genome, such as papillomaviruses and polyomaviruses, awaits further investigation.

HMGB1 is almost ubiquitous and only 10 times less abundant than core histones, at 10^6^ molecules per typical mammalian cell [71]. Oppositely, cccDNA and HBx protein exist in the infected hepatocytes at a low or even hardly detectable level [9,23]. In our study we have observed a fine intracellular interplay of HMGB1 and HBx protein, where HBx counteracts inhibitory effect of HMGB1 on cccDNA transcription by either displacing or preventing HMGB1 binding to the cccDNA. Even though HBx has not been shown to directly bind cccDNA, it undoubtedly upregulates its transcriptional activity *via* hijacking host factors [20], and potentially mediates spatial hindrance for HMGB1-cccDNA physical interaction at the intranuclear compartment(s) where the cccDNA transcription events take place. Thus, the stoichiometry of HBx-HMGB1 interaction that confers the transcription activity of cccDNA remains to be defined in future study.

During primary HBV infection, the nascent cccDNA may be immediately targeted by the pre-existing intranuclear HMGB1, and then actively counteracted by *de novo* synthesized HBx protein imported to the nucleus. Recently, it has been shown that HBx is expressed early after infection and has a ~3 hours half-life in cell culture model [72], hence, such interaction may happen at the initial steps of HBV infection. In an alternative scenario, during *de novo* infection, HBx protein or HBx mRNA might be delivered along with uncoated rcDNA, prior cccDNA formation and its first round of transcription, so that HBx can start preventing HMGB1 binding to cccDNA even earlier and keep doing that over the course of infection. The exact mechanisms and the order of HMGB1-HBx counteraction events in cccDNA chromatin regulation are subjects for further study.

Inside cell nucleus, HMGB1 serves as a structural non-histone protein, binding and bending DNA duplex. The exact mechanisms underlying DNA-binding and epigenetic regulation of chromatin by HMGB1 remain largely unknown. It has been reported that HMGB1 counteracts chromatin condensation-associated linker histone H1 to compete for the same inter-nucleosomal binding sites and fluidize chromatin to increase accessibility by transcription factors [73]. In our study, HBx-deficient cccDNA was highly enriched with both H1 and HMGB1 (Figs S6 and 5), so for cccDNA, the HMGB1-H1 interaction, if any, is in an agonistic relationship, where HMGB1 helps H1 to “lock” the chromatin as in an epigenetic silencing model. This mode of action is consistent with a previous study demonstrating that chromatin remodeling by HMGB1 and H1 silenced tumor necrosis factor-α (TNFα) gene promoter during endotoxin tolerance [74]. Furthermore, another study showed that HMGB1 silences a tumor growth and metastasis repressor semaphorin SEMA3A *via* directly binding to its genomic locus, promoting heterochromatin formation and decreased occupancy of acetylated histones [75]. Thus, even though HMGB1 is known to participate in kinking linear DNA to potentially increase its accessibility to transcription and DNA repair machinery, it is also considered as a gene silencer, for instance, irreversibly binding to DNA with the formation of solid complexes with preferences to underacetylated chromatin during apoptosis events [57].

Although HMGB1 is mostly considered to bind DNA non-specifically either by recognizing secondary structure of DNA molecules or its forms such as minicircles [58], there is potential evidence of a DNA-binding site specificity for HMGB1. It has been shown that HMGB1 upregulates TNFα gene *via* binding to the receptor activator for nuclear factor-kappa B ligand (RANKL)-responsive sequence (CCG AGA CAG AGG TGT AGG GCC), spanning from -157 to -137 bp of the 5’-flanking region of the TNFα gene [76]. Interestingly, this motif shares similarity of its 14–15 out of 21 nt fragment with the antisense strand (nt 731–710) of HBV genomes across genotype A-H. This and other potential HMGB1-binding site(s) on cccDNA can be investigated by electrophoretic mobility shift assay (EMSA) or HMGB1-specific ChIP-seq assay in future study. In addition, the ChIP-seq assay will also reveal the potential alteration of HMGB1-binding sites on host genome in response to HBx.

**Fig 11:**
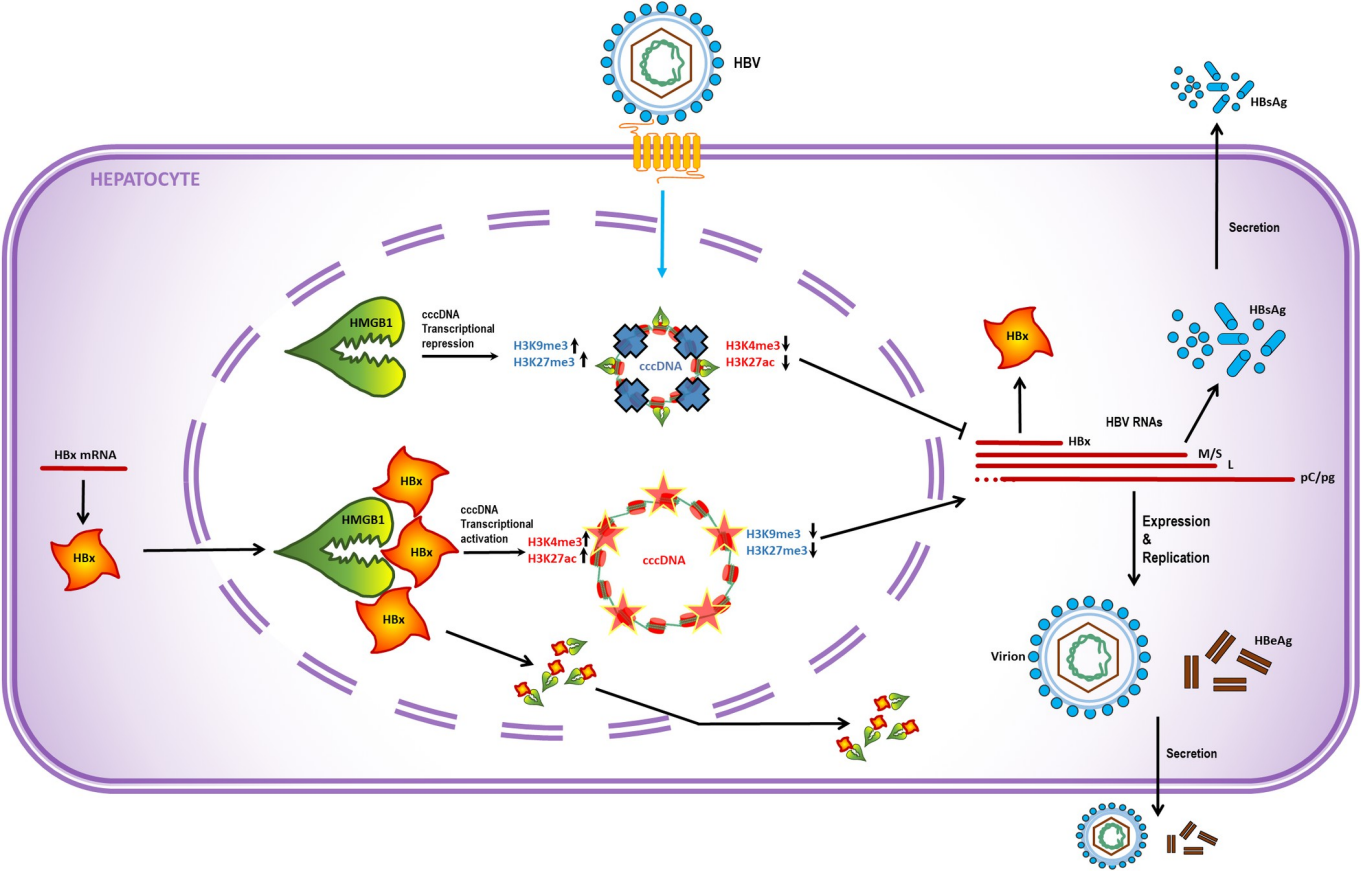
Mode of action of HMGB1-HBx interplay. Newly formed cccDNA is targeted by preexisting intranuclear host restriction factor HMGB1, which mediates epigenetic silencing of cccDNA minichromosome. Transcriptionally repressed cccDNA lacks active histone PTMs (represented by H3K27ac and H3K4me3) and enriched with repressive histone PTMs (represented by H3K27me3 and H3K9me3). Viral HBx protein counteracts HMGB1 to prevent its association with cccDNA and induce partial nuclear-cytoplasmic translocation of HMGB1, thereby conferring an active epigenetic state of cccDNA transcription.

Taken together, our study revealed that host protein HMGB1 targets the HBV cccDNA minichromosome and induces epigenetic silencing of viral transcription, which could be antagonized by viral HBx protein (Fig 11). Fine mechanisms of HMGB1-associated viral chromatin tuning *via* HBV cccDNA epiproteome modulation are subject to further investigation. It is also of interest to investigate the potential HBx-dependent relationships between HMGB1 and other reported cccDNA restriction factors, including Smc5/6 complex, in cccDNA epigenetic regulation. Chronic HBV persistence is sustained by the cccDNA molecule which cannot be eradicated by any of currently known drugs. Thus, uncovering complex mechanisms of chromatin condensation of cccDNA and principles of epigenetic regulation of cccDNA episome in its interplay with host factors such as HMGB1 protein could allow us to elaborate new strategies for addressing the unmet clinical need of functional cure of HBV.

## Materials and methods

### Plasmids and transfection

HBV (genotype D, subtype *ayw*) replication competent plasmid pCMVHBV, which the transcription of viral pgRNA is governed by the human cytomegalovirus immediate-early (CMV-IE) promoter, was described previously [77]. The plasmid pTREHBV-HAe transcribes pgRNA under the control of tetracycline-inducible CMV promoter, which has been previously used together with plasmid pTet-Off (Clontech) to generate the HepBHAe82 stable cell line that expresses the cccDNA-dependent hemagglutinin (HA)-tagged HBV e antigen (HA-HBeAg) [37]. To generate HBx-null HBV plasmids, a point mutation (C1397T) was introduced to the HBV sequence in pCMVHBV and pTREHBV-HBe by using Q5 Site-Directed Mutagenesis Kit (NEB) (S2 Table), giving rise to plasmid pCMVHBVΔx and pTREHBVΔx-HBe, respectively. The C1397T mutation induces HBx ORF premature termination *via* mutation of HBx 8^th^ amino acid codon CAA (Q, Gln) to stop codon (TAA) without altering the amino acid sequence of the overlapping HBV polymerase gene. Plasmid FLAG-HBx expressing N-terminally FLAG-tagged HBx protein was kindly provided by Dr. Michael Bouchard (Drexel University) [78]. Retroviral vector pWZL-Blast and packaging plasmids pUMCV3 and VSV-G were described previously [79]. The ORF sequence of wt HBx was cloned into retroviral vector pWZL-Blast to generate plasmid pWZL-HBx. To construct plasmid His-HMGB1 expressing the C-terminally 6×His-tagged HMGB1, the designed ORF of His-tagged HMGB1 was chemically synthesized by Genscript and cloned into pcDNA3.1/V5-His TOPO TA expression vector (K480001, Invitrogen). Plasmid transfection into cells was performed using Lipofectamin 3000 (L3000150, Invitrogen).

### Cell cultures

HepG2 and 293T cell lines were maintained in F12/DMEM media (SH30023.01, HyClone) with 10% heat-inactivated FBS (S10350, Atlanta Biologicals), 100 U/ml penicillin, 100 μg/ml streptomycin.

The HepG2-based inducible HBV stable cell lines, including HepAD38 and HepBHAe82 (and sibling clones HepBHAe1 and HepBHAe45), were maintained in the same way as HepG2 but with the addition of 1 μg/ml tetracycline (tet) and 500 μg/ml G418 [37,80,81]. For induction of HBV production, the media was changed to analogous but tetracycline-free one with 2% heat-inactivated FBS for indicated time duration. HepG2-H1.3Δx cell line constrictively replicating HBV in the absence of HBx has been described previously [19].

HepG2-NTCP cells were maintained in the same way as HepG2 with the addition of 8 μg/ml Blasticidin (A1113903, Life technologies) [82]. The primary human hepatocyte (PHH)-derived PXB-cells freshly isolated from PXB mice with humanized liver were purchased from PhoenixBio and maintained in manufacturer’s modified dHCGM media (PhoenixBio, Canada) [83].

### HepBHAeΔx cell line establishment

HepG2 cells were co-transfected with pTREHBVΔx-HAe and pTet-Off with 7:1 molar ratio, followed by selection with 500 μg/ml G418 in the presence of 1 μg/ml tet. G418-resistant colonies were picked and expanded into cell lines. To determine HBV-positive cell lines, HBV DNA replication was induced by culturing candidate cell lines in tet-free medium for 6 days, then the intracellular HBV DNA positivity was assessed by dot blot hybridization assay as previously described [37]. The obtained HBV positive cell lines were further assessed for their tet-inducible (tet-off) HBV DNA replication in the presence and absence of tet by conventional Southern blot (S3 Fig). Two tet-inducible cell clones with high levels of HBV DNA replication were designated HepBHAeΔx67 and 114 cell lines. The maintenance and induction of HepBHAeΔx cells were performed in the same way as HepBHAe82 cells.

To transcomplement HBx expression in HepBHAeΔx67 cells, pWZL-HBx was co-transfected with pUMCV3 and VSV-G into 293T cells to package retroviral HBx-expression particles, which were then used to transduce HepBHAeΔx67 cells, followed by Blasticidin (8 μg/ml) selection in the presence of tet. The Blasticidin-resistant cells were pooled and expanded into to HepBHAeΔx67+HBx cells.

### HBV infection in cell cultures

HBV wild-type infectious particles and HBx-null HBVΔx virus particles were collected from the supernatant of HepAD38 and HepG2-H1.3Δx cells, respectively, and the virion genome equivalents (VGE) were determined as previously described [19,82]. The infection of HepG2-NTCP cells and PXB-cells by HBV particles was conducted as previously published [84].

### HBV nuclei acids analyses

HBV total RNA, cytoplasmic core DNA, and total Hirt DNA (contain deproteinated rcDNA and cccDNA) were extracted and subjected to Northern and Southern blotting as described previously [85,86]. Hybridization signals were recorded on a phosphorimager screen and scanned by the Typhoon FLA-7000 imager (GE Healthcare). The reverse transcription PCR (RT-PCR) assays of HBV precore (pC) mRNA and cellular β-actin mRNA were performed using primers listed in S2 Table and the RT-PCR products were detected by agarose gel electrophoresis according to our previous publications [38,87].

For HBV cccDNA qPCR assay, the Hirt DNA samples were heat-denatured and subsequently treated with Plasmid-safe ATP-dependent DNase (PSAD) (Epicentre), then quantified by qPCR and normalized to mitochondrial DNA according to our previous publication [88]. qPCR for cccDNA and total Hirt HBV DNA was performed using FastStart Essential DNA Probes Master kit (06402682001, Roche); mitochondrial COX3 qPCR was done with FastStart Essential DNA Green Master kit (06402712001, Roche) (S2 Table).

To quantify HBV pC mRNA, total RNA was purified using RNeasy Mini Kit (74106, Qiagen), supplemented with QIAshredder columns (79656, Qiagen). One-step RT-qPCR for HBV pC mRNA and cellular GAPDH mRNA qPCR was performed on Roche LightCycler 96 System (Roche) with LightCycler RNA Master Hydrolysis Probes 2.7× kit (04991885001, Roche) and primers and probes listed in S2 Table.

### HBV antigens detection

The HA-HBeAg in culture fluid was detected by chemiluminescence immunoassay (CLIA) according to a previously published protocol [37]. The detection of authentic HBeAg was performed by the commercial HBeAg ELISA kit (CSB-E13557h, Cusabio) following the manufacturer’s manual. HA-HBeAg CLIA signals and HBeAg ELISA OD450nm values were read by the Synergy HTX plate reader (BioTek). The intracellular HBV core antigen (HBcAg) were detected by immunofluorescence microscopy as previously described [84]

### Western immunoblotting

Whole cell lysate samples were prepared using Laemmli buffer, resolved in precast SDS-PAGE gels (Life Technologies), and transferred onto Immobilon-PSQ Membranes (Millipore). The membranes were incubated in buffers from the WesternBreeze Blocker/Diluent kit (Life Technologies) and probed with corresponding primary antibodies (S3 Table). Bound antibodies were detected by IRDye secondary antibodies using the Li-COR Odyssey Fc system (Li-COR).

### Chromatin immunoprecipitation coupled with qPCR (ChIP-qPCR)

ChIP-qPCR was performed on approximately 2×10^6^ cells per reaction fixed with 1% formaldehyde using the ChIP-IT Express Kit with protein G magnetic beads (53008, Active Motif) according to the manufacturer’s protocol. Chromatin was sheared using EpiShear ultrasound sonicator (53051, Active Motif), with delivery of 50–250 Joules in dependence of cell line for obtaining chromatin fragments with DNA length around 500–1500 bp long. The sheared chromatin was precipitated with 5 μg of specific or non-immune serum (NIS) IgG control antibodies (S3 Table). After the removal of formaldehyde crosslinks, immunoprecipitated chromatin was deproteinized with 10 μg/ml proteinase K. The input control and immunoprecipitated DNA fractions were purified with QIAquick PCR Purification Kit (28106, Qiagen), and served as templates in cccDNA qPCR amplification. Occupancy of the specific protein on cccDNA was expressed in percentage (%) of input using -ΔCq methods.

### Nuclear HBV core protein co-immunoprecipitation and LC-MS/MS analysis

1×10^8^ HepAD38 and HepBHAeΔx67 cells were induced for HBV replication for 14 days. The nuclear fractions of the cells, which contained cccDNA, were isolated using the Nuclear Extraction Kit (10009277, Cayman Chemical) and subsequently incubated with a 1:50 dilution of anti-HBc antibodies (B0586, Dako) (S3 Table) for 3 hours at 4°C under constant and gentle agitation. Then, the HBc complex pulldown by Protein G magnetic beads and intensive washing were then performed accordingly to Universal Magnetic Co-IP Kit manufacturer’s instruction (54002, Active Motif). The beads-bound proteins were reduced, alkylated, and trypsin digested directly on beads, and the subsequent peptides were identified with liquid chromatography interfaced with a hybrid quadrupole-Orbitrap mass spectrometer (ThermoFisher Q Exactive HF Orbitrap LC-MS/MS system) at the Purdue University Proteomics Facility. Database search against Uniprot human database was conducted using MaxQuant to identify the probable source proteins of the peptides.

### Compounds

HMGB1 inhibitor Glycyrrhizic acid ammonium salt (glycyrrhizin) from licorice root (*Glycyrrhiza glabra* L.) was purchased from Millipore-Sigma (50531-10G) and dissolved in 1×DPBS to make 20 mM stock. HBV replication inhibitor Lamivudine (3TC) was purchased from Selleck Chemicals and dissolved in DMSO as 10 mM stock.

### HMGB1 knockdown

Stable knockdown of HMGB1 in HepG2-NTCP and HepBHAeΔx67 cells was performed using lentiviral-shHMGB1 particles from a customized Mission lentiviral shRNA library (CSTVRS 090513023MN, P2/E3, TRCN000018934, Sigma) [88], in presence of 10 μg/ml of Polybrene (sc-134220, Santa Cruz Biotechnology), followed by 3 μg/ml Puromycin (A11138-03, Life technologies) selection and knockdown validation with Western immunoblotting. Control knockdown cells were generated similarly by Control shRNA Lentiviral Particles-A (sc-108080, Santa Cruz Biotechnology).

### Co-immunoprecipitation (Co-IP)

The Co-IP of HBx and endogenous HMGB1 protein were performed in cells transfected by FLAG-HBx plasmid using Anti-HMGB1/HMG1 Magnetic Beads Immunoprecipitation (IP) Kit (MB100930-T46, Sino Biologicals) followed by Western immunoblotting.

### Statistical analysis

Data are provided as the mean ± standard error (SEM) or standard deviation (SD) of the mean. Statistical significance was considered with p value less than 0.05 (* p<0.05; ** p<0.01; *** p<0.001), and calculations and graphs were generated using GraphPad Prism 9.0.

## Supporting information

S1 Fig HBx protein activates plasmid-based HBV transcription and replication. (A) HepG2 cells in 12-well-plate were co-transfected with 0.8 μg of control vector and equal amount of pCMVHBV (lane 1) or pCMVHBVΔx (lane 2), or co-transfected with 0.8 μg of each pCMVHBVΔx and FLAG-HBx (lane 3), for 5 days. HBV total RNA, cytoplasmic core DNA, and FLAG-tagged HBx expression were analyzed by Northern blotting hybridization, Southern blotting hybridization, and anti-FLAG Western immunoblotting, respectively. (B) HepG2 cells in 12-well-plate were co-transfected with 0.8 μg of control vector and equal amount of pCMVHBV (lane 1) or pCMVHBVΔx (lane 2), or co-transfected with 0.8 μg of pCMVHBVΔx plus increasing amount of FLAG-HBx with mass ratio of 1,000:1, 100:1, 10:1, 1:1 (lanes 3–6), control vector was supplemented to normalize the total amount of the transfected plasmids to 1.6 μg. HBV total RNA, cytoplasmic core DNA, and HBx expression were analyzed by Northern blotting hybridization, Southern blotting hybridization, and anti-HBx Western immunoblotting, respectively.
(TIF)

S2 Fig Schematic design of the HBx-null tet-inducible HepBHAeΔx cell line. (A) The viral transgene contains a 1.1 overlength HBV genome under the control of tet-CMV promoter. The start codon (ATG) of preCore (pC) was mutated at the 5’ end of HBV DNA, with the second one unchanged at the 3’ redundancy. The HA-tag-containing fragment (shown in green) was inserted into the pC domain upstream of the start codon of core ORF. A point nucleotide mutation (C1397T, shown in red) is introduced to the HBx ORF and terminates its expression. The transgene also contains two tandem stop codons in the small surface (S) ORF to prevent viral envelope protein expression. (B) In the presence of tTA expression, upon removal of tet from culture medium, pgRNA is transcribed and core (C) and polymerase (pol) are produced, resulting in pgRNA packaging and (C-D) reverse transcription of pgRNA to rcDNA in cytoplasm. (E) A portion of rcDNA is recycled to nucleus and converted into cccDNA template, in which the HA-pC ORF is restored, giving rise to HA-pC mRNA, if cccDNA is under transcriptionally active stage, and (F) pgRNA for *de novo* viral replication. (G) HA-pC is translated from HA-pC mRNA and processed into secreted HA-HBeAg in the newly formed cccDNA-dependent manner and thus serves as a semi-quantitative marker for cccDNA expression level in ELISA. DR: direct repeat sequence. CTD: C-terminal domain.
(TIF)

S3 Fig Establishment of HepBHAeΔx67 cell line. (A) HepG2 cells were co-transfected with pTet-Off and pTREHBV-HAe or pTREHBV**Δ**x-HAe in mass ratio of 1:2 for 5 days, HBV total RNA and cytoplasmic core DNA were analyzed by Northern blot and Southern blot, respectively. (B) Four candidate HepBHAe**Δ**x clones #55, #67, #99 and #114 were assessed for their tet-off inducibility and HBV replication levels. Cell clones were cultured in the presence or absence of tet for 6 days and subjected to HBV core DNA Southern blot assay.
(TIF)

S4 Fig Restoration of HBV cccDNA transcription in HepBHAeΔx67 cells by HBx transcomplementation. (A) HepBHAe82, HepBHAeΔx67 and HepBHAeΔx67+HBx cells were induced for 14 days in the absence of tet, cccDNA-dependent pC mRNA was detected by RT-PCR. β-actin mRNA RT-PCR served as control. (B) Supernatant HA-HBeAg signals in HepBHAeΔx67 and HepBHAeΔx67+HBx cells at day 10 and 14 post-induction were detected by CLIA and normalized to cccDNA qPCR quantification.
(TIF)

S5 Fig Comparative epigenetic profiling of cccDNA among HepBHAeΔx67 cell line and other HepBHAe82 sibling clones. HepBHAeΔx67 cells, HepBHAe82 and sibling clones HepBHAe1 and HepBHAe45 cells were induced for HBV replication for 14 days, the association of (A) active histone PTM H3K27ac and (B) repressive histone PTM H3K27me3 with cccDNA was assessed by ChIP-qPCR (% of input; mean ± SEM, n = 3). *p<0.05, **p<0.01, ***p<0.001.
(TIF)

S6 Fig The enriched repressive epigenetic markers on cccDNA in the absence of HBx. HepAD38 (HBx-positive) and HepBHAeΔx67 (HBx-null) cells were induced for HBV replication for 14 days, the association of H3K9me2, SETDB1, SETDB2, and histone H1 with cccDNA was assessed by ChIP-qPCR (% of input; mean ± SEM, n = 3). ns: not significant; **p<0.01,
(TIF)

S7 Fig Principal scheme of the cccDNA chromatin immunoprecipitation and mass spec (IP-MS) assay. (A) cccDNA minichromosomes from induced HepAD38 and HepBHAeΔx67 cells were immobilized on the surface of magnetic beads *via* HBc-specific capture by antibodies. The immunoprecipitated preparations underwent on-beads digestion and peptide identification by liquid chromatography tandem mass spectrometry (LC-MS/MS). (B) The identified protein hits were subgrouped into three categories, depicted on the Venn diagram for the non-histone host POIs: wt HBV-specific hits (green), HBx-deficient HBV-specific hits (red), and shared hits (yellow). Full proteins names are listed in the S1 Table. HBc: HBV core protein; αHBc: anti-HBc antibody; beads: Protein G covered magnetic beads; POI: protein of interest.
(TIF)

S8 Fig HBx reduces HMGB1 enrichment on cccDNA. HepBHAe82, HepBHAeΔx67 and HepBHAeΔx67+HBx cells were induced for 14 days, the association of HMGB1 with cccDNA was assessed by ChIP-qPCR (% of input; mean ± SEM, n = 3). BDL: below detection limit.
(TIF)

S9 Fig Upregulation of cccDNA transcription by HMGB1 inhibitor glycyrrhizin in the absence of HBx. HepBHAeΔx67 cells were induced in tet-free medium for 8 days, followed by mock (1×DPBS) or glycyrrhizin (10 μM) treatment for 4 days in the presence of tet and 3TC (10 μM). HBV pC mRNA was quantified by RT-qPCR and normalized to GAPDH mRNA and cccDNA (mean ± SEM, n = 3), *p<0.05.
(TIF)

S1 Table Nuclear HBc pulldown host protein hits. (PDF)

S2 Table List of oligonucleotides used in the study. (PDF)

S3 Table List of antibodies used in the study. (PDF)
