# Supplementary figures and images for: Hepatitis B virus X protein counteracts high mobility group box 1 protein-mediated epigenetic silencing of covalently closed circular DNA
Source: PLoS Pathog. 2022 Jun 9;18(6):e1010576. doi: 10.1371/journal.ppat.1010576 (PMC9182688; doi:10.1371/journal.ppat.1010576)

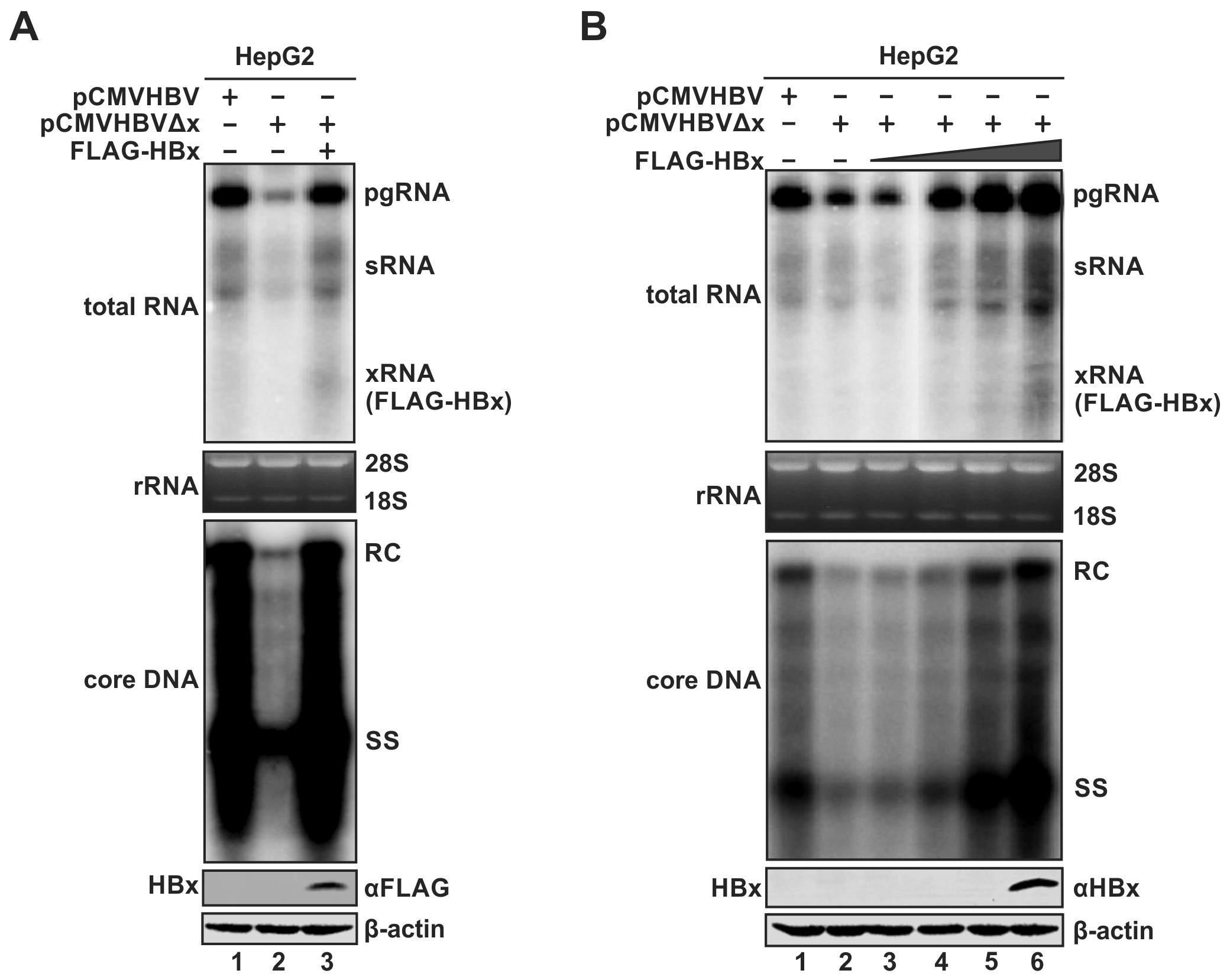

Supplement: S1 Fig — (A) HepG2 cells in 12-well-plate were co-transfected with 0.8 μg of control vector and equal amount of pCMVHBV (lane 1) or pCMVHBVΔx (lane 2), or co-transfected with 0.8 μg of each pCMVHBVΔx and FLAG-HBx (lane 3), for 5 days. HBV total RNA, cytoplasmic core DNA, and FLAG-tagged HBx expression were analyzed by Northern blotting hybridization, Southern blotting hybridization, and anti-FLAG Western immunoblotting, respectively. (B) HepG2 cells in 12-well-plate were co-transfected with 0.8 μg of control vector and equal amount of pCMVHBV (lane 1) or pCMVHBVΔx (lane 2), or co-transfected with 0.8 μg of pCMVHBVΔx plus increasing amount of FLAG-HBx with mass ratio of 1,000:1, 100:1, 10:1, 1:1 (lanes 3–6), control vector was supplemented to normalize the total amount of the transfected plasmids to 1.6 μg. HBV total RNA, cytoplasmic core DNA, and HBx expression were analyzed by Northern blotting hybridization, Southern blotting hybridization, and anti-HBx Western immunoblotting, respectively. (TIF) [file ppat.1010576.s001.tif]

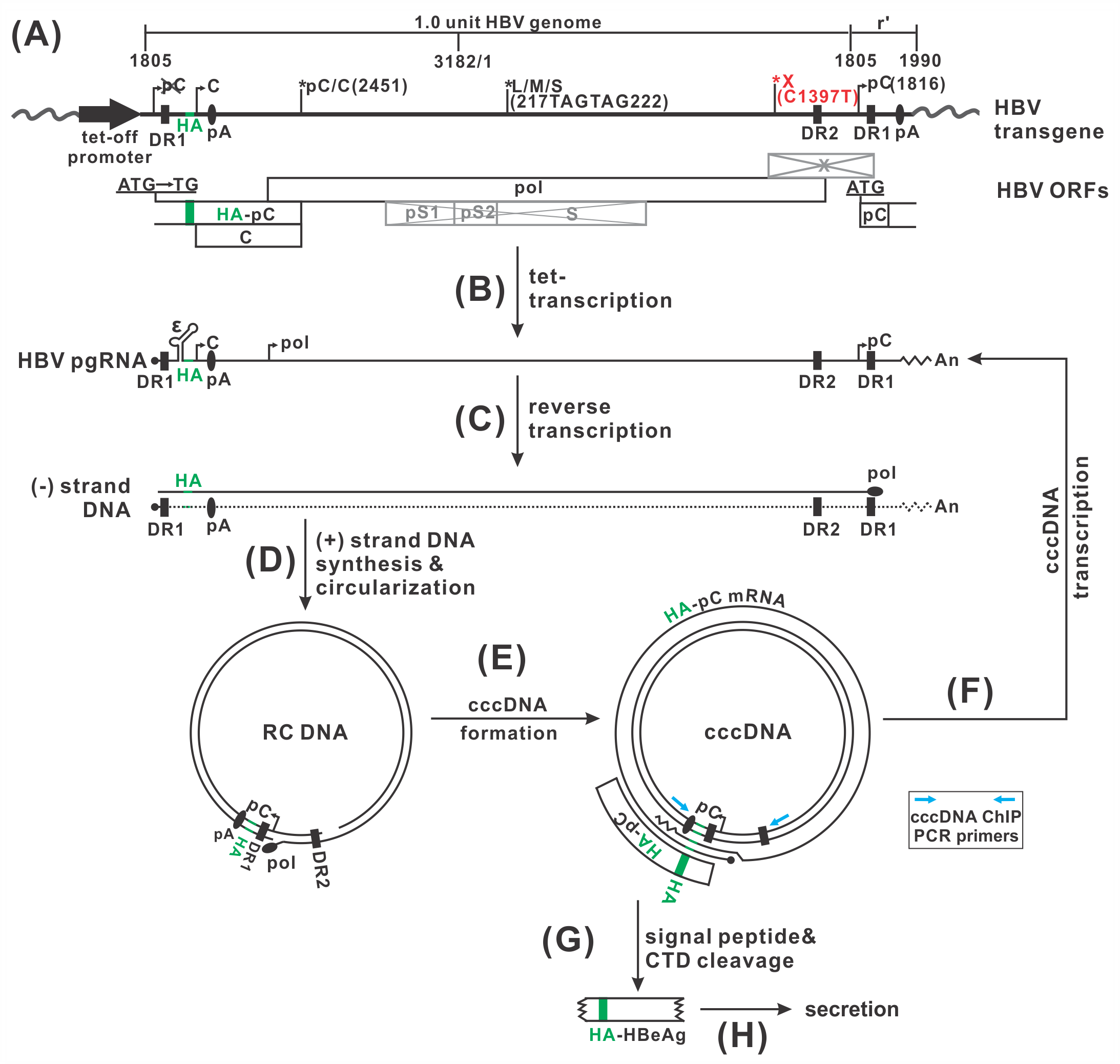

Supplement: S2 Fig — (A) The viral transgene contains a 1.1 overlength HBV genome under the control of tet-CMV promoter. The start codon (ATG) of preCore (pC) was mutated at the 5’ end of HBV DNA, with the second one unchanged at the 3’ redundancy. The HA-tag-containing fragment (shown in green) was inserted into the pC domain upstream of the start codon of core ORF. A point nucleotide mutation (C1397T, shown in red) is introduced to the HBx ORF and terminates its expression. The transgene also contains two tandem stop codons in the small surface (S) ORF to prevent viral envelope protein expression. (B) In the presence of tTA expression, upon removal of tet from culture medium, pgRNA is transcribed and core (C) and polymerase (pol) are produced, resulting in pgRNA packaging and (C-D) reverse transcription of pgRNA to rcDNA in cytoplasm. (E) A portion of rcDNA is recycled to nucleus and converted into cccDNA template, in which the HA-pC ORF is restored, giving rise to HA-pC mRNA, if cccDNA is under transcriptionally active stage, and (F) pgRNA for de novo viral replication. (G) HA-pC is translated from HA-pC mRNA and processed into secreted HA-HBeAg in the newly formed cccDNA-dependent manner and thus serves as a semi-quantitative marker for cccDNA expression level in ELISA. DR: direct repeat sequence. CTD: C-terminal domain. (TIF) [file ppat.1010576.s002.tif]

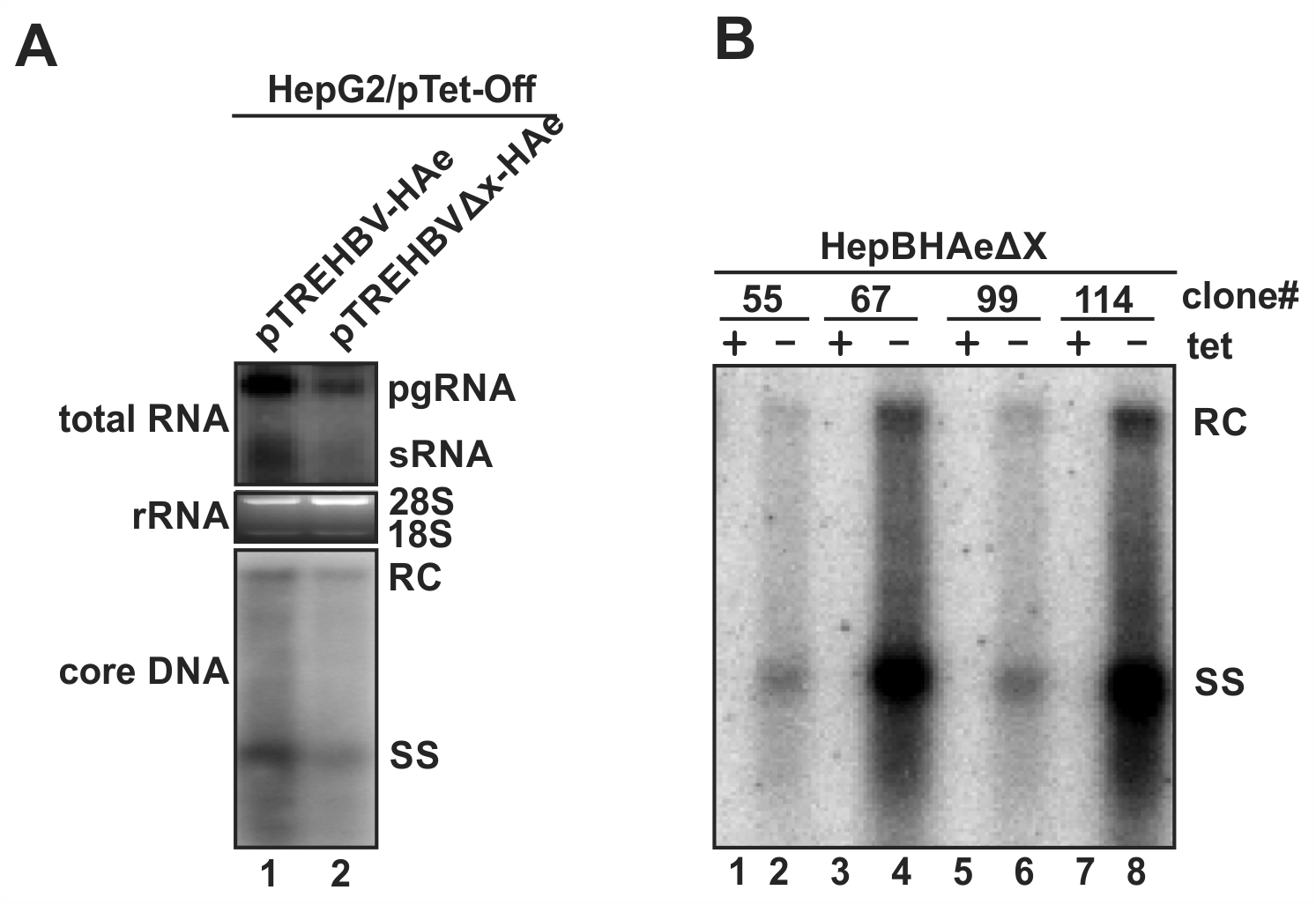

Supplement: S3 Fig — (A) HepG2 cells were co-transfected with pTet-Off and pTREHBV-HAe or pTREHBVΔx-HAe in mass ratio of 1:2 for 5 days, HBV total RNA and cytoplasmic core DNA were analyzed by Northern blot and Southern blot, respectively. (B) Four candidate HepBHAeΔx clones #55, #67, #99 and #114 were assessed for their tet-off inducibility and HBV replication levels. Cell clones were cultured in the presence or absence of tet for 6 days and subjected to HBV core DNA Southern blot assay. (TIF) [file ppat.1010576.s003.tif]

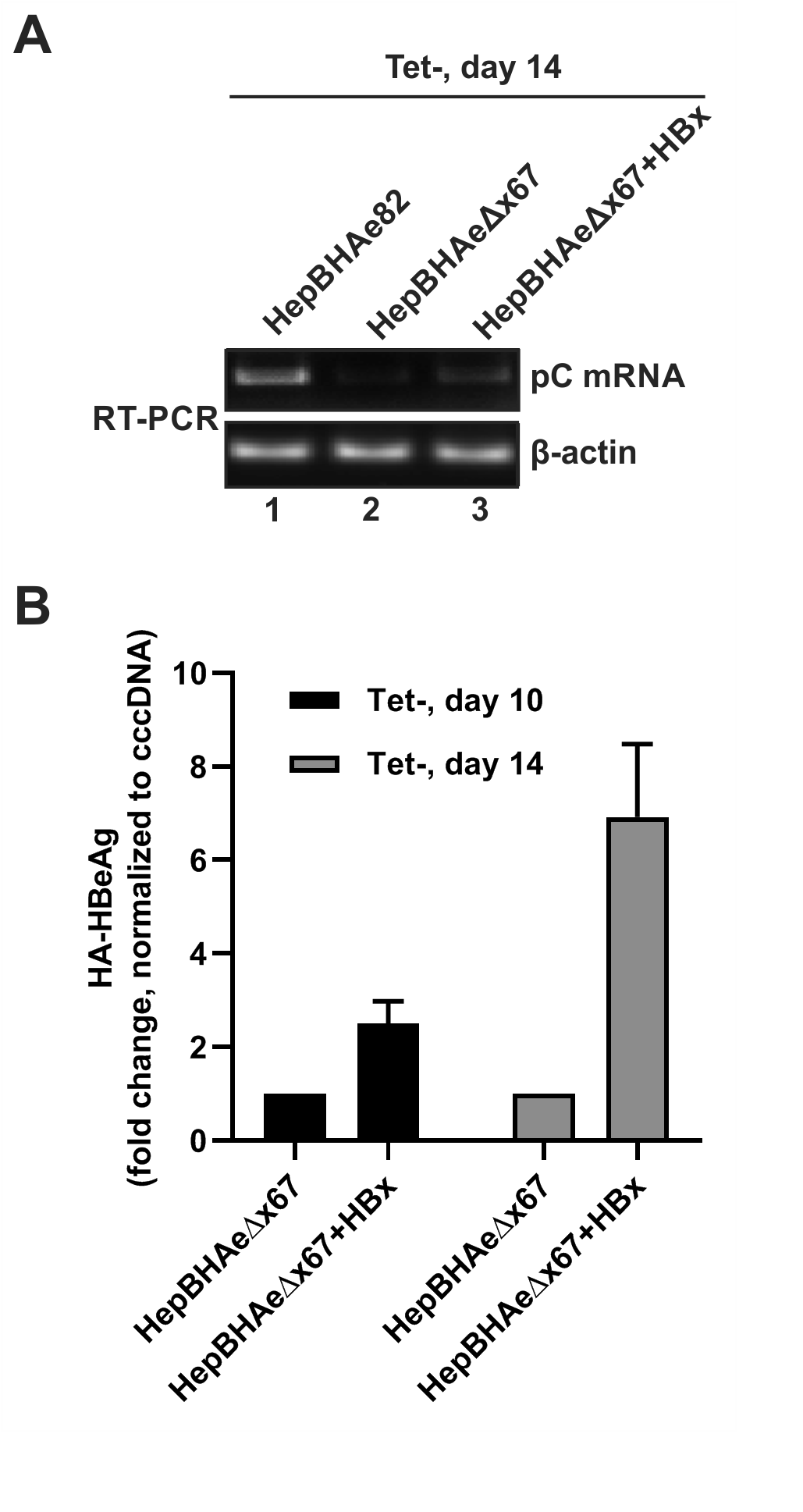

Supplement: S4 Fig — (A) HepBHAe82, HepBHAeΔx67 and HepBHAeΔx67+HBx cells were induced for 14 days in the absence of tet, cccDNA-dependent pC mRNA was detected by RT-PCR. β-actin mRNA RT-PCR served as control. (B) Supernatant HA-HBeAg signals in HepBHAeΔx67 and HepBHAeΔx67+HBx cells at day 10 and 14 post-induction were detected by CLIA and normalized to cccDNA qPCR quantification. (TIF) [file ppat.1010576.s004.tif]

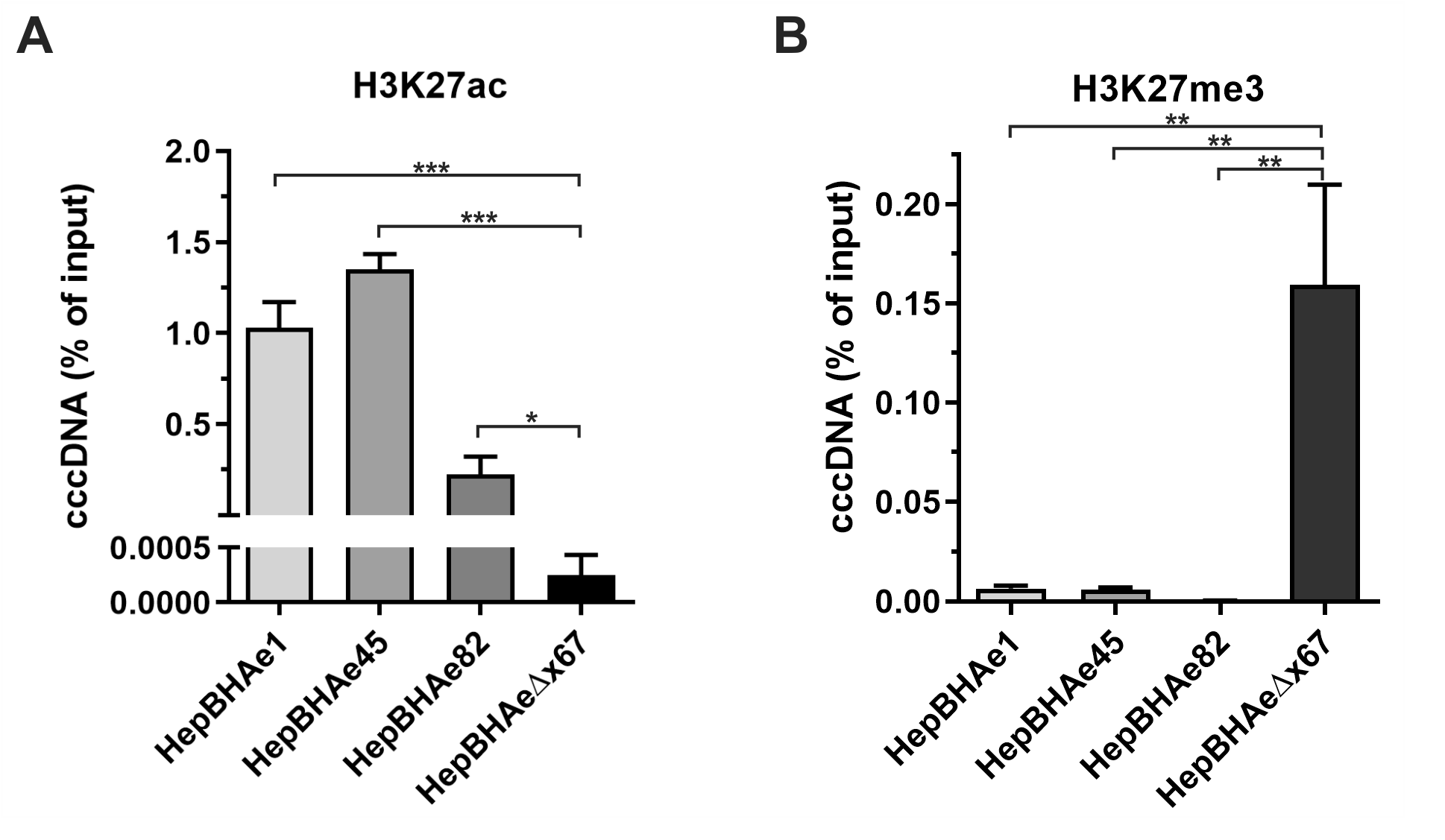

Supplement: S5 Fig — HepBHAeΔx67 cells, HepBHAe82 and sibling clones HepBHAe1 and HepBHAe45 cells were induced for HBV replication for 14 days, the association of (A) active histone PTM H3K27ac and (B) repressive histone PTM H3K27me3 with cccDNA was assessed by ChIP-qPCR (% of input; mean ± SEM, n = 3). *p<0.05, **p<0.01, ***p<0.001. (TIF) [file ppat.1010576.s005.tif]

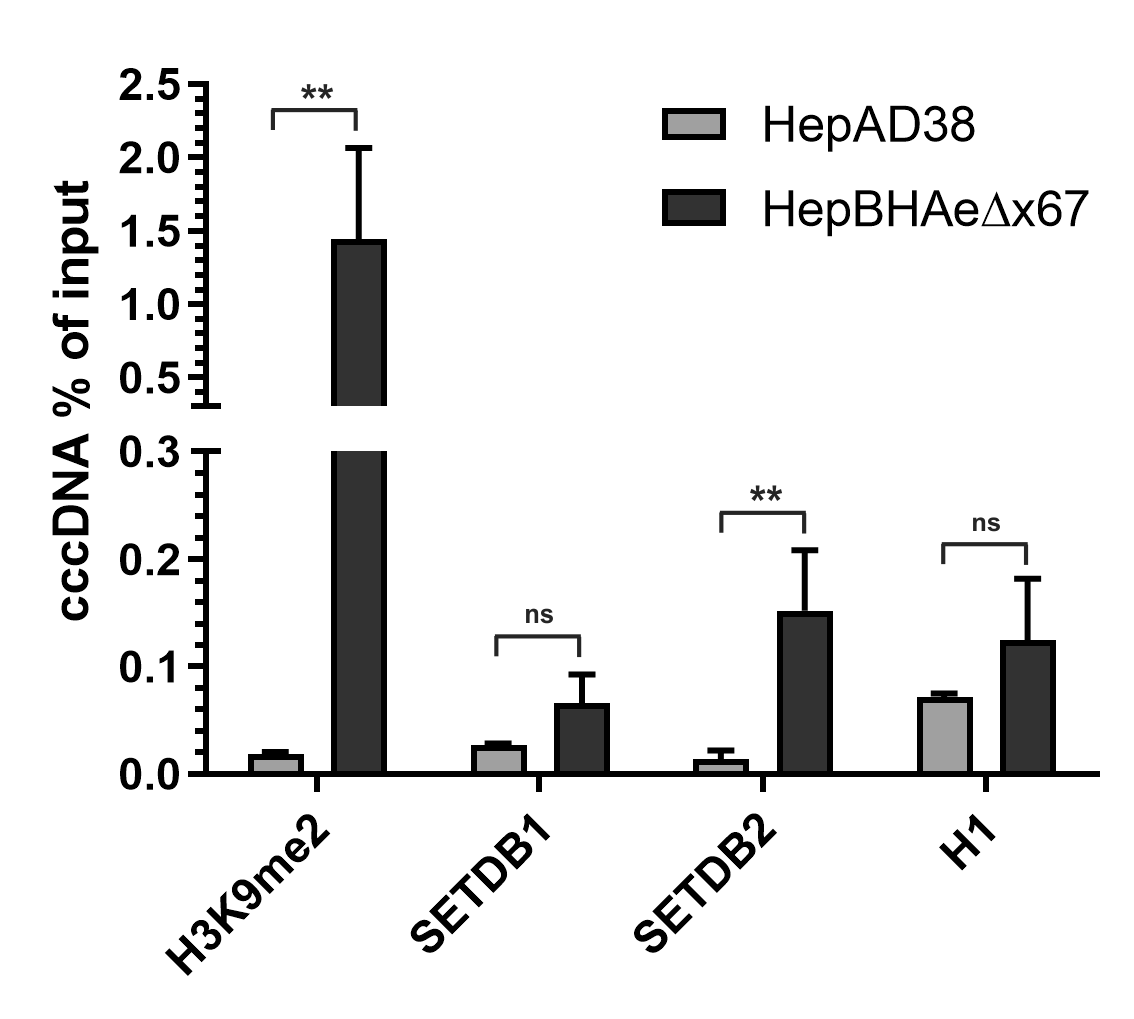

Supplement: S6 Fig — HepAD38 (HBx-positive) and HepBHAeΔx67 (HBx-null) cells were induced for HBV replication for 14 days, the association of H3K9me2, SETDB1, SETDB2, and histone H1 with cccDNA was assessed by ChIP-qPCR (% of input; mean ± SEM, n = 3). ns: not significant; **p<0.01, (TIF) [file ppat.1010576.s006.tif]

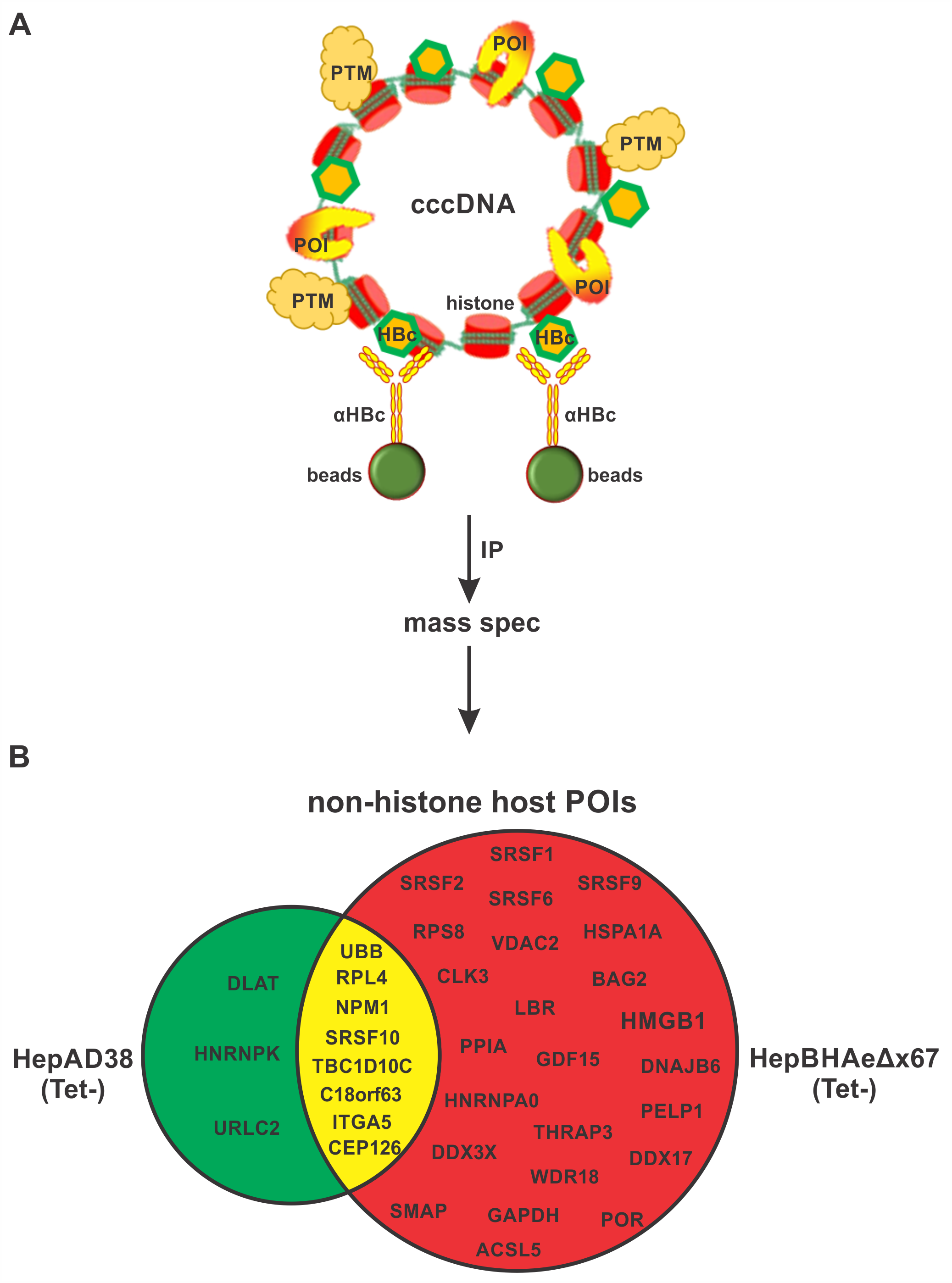

Supplement: S7 Fig — (A) cccDNA minichromosomes from induced HepAD38 and HepBHAeΔx67 cells were immobilized on the surface of magnetic beads via HBc-specific capture by antibodies. The immunoprecipitated preparations underwent on-beads digestion and peptide identification by liquid chromatography tandem mass spectrometry (LC-MS/MS). (B) The identified protein hits were subgrouped into three categories, depicted on the Venn diagram for the non-histone host POIs: wt HBV-specific hits (green), HBx-deficient HBV-specific hits (red), and shared hits (yellow). Full proteins names are listed in the S1 Table. HBc: HBV core protein; αHBc: anti-HBc antibody; beads: Protein G covered magnetic beads; POI: protein of interest. (TIF) [file ppat.1010576.s007.tif]

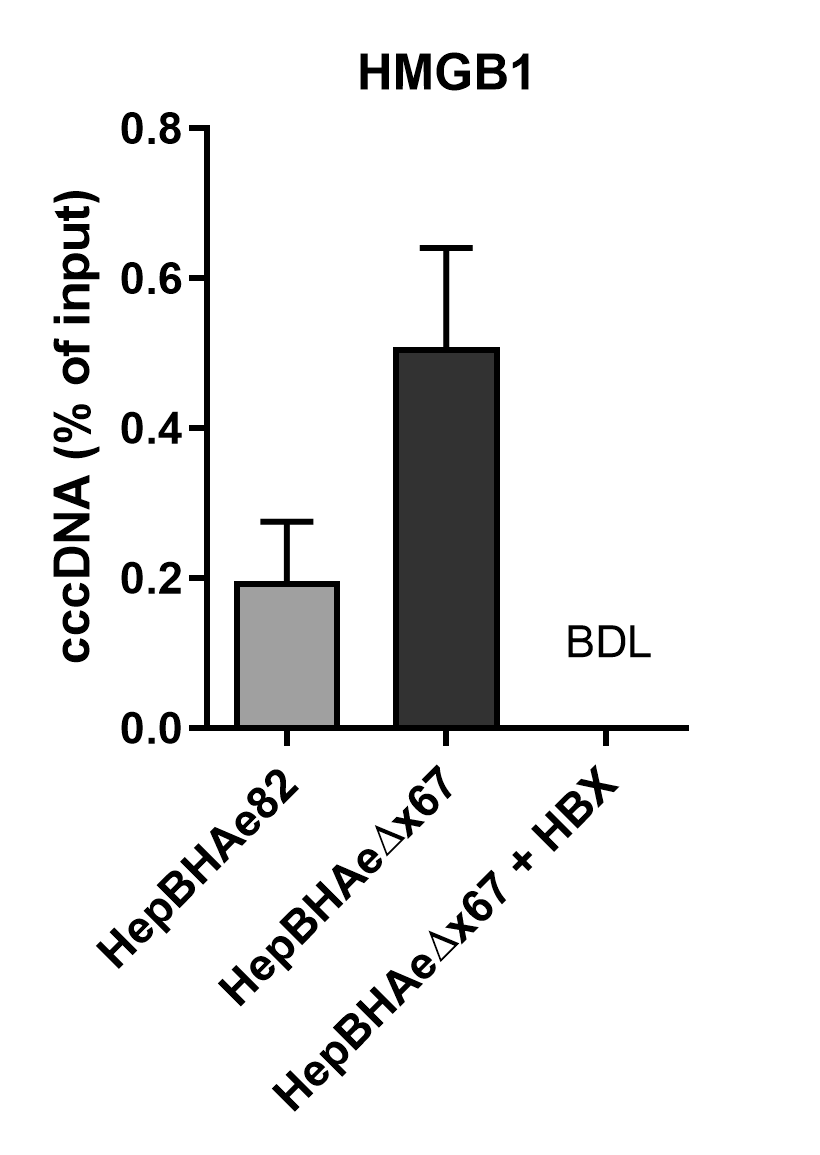

Supplement: S8 Fig — HepBHAe82, HepBHAeΔx67 and HepBHAeΔx67+HBx cells were induced for 14 days, the association of HMGB1 with cccDNA was assessed by ChIP-qPCR (% of input; mean ± SEM, n = 3). BDL: below detection limit. (TIF) [file ppat.1010576.s008.tif]

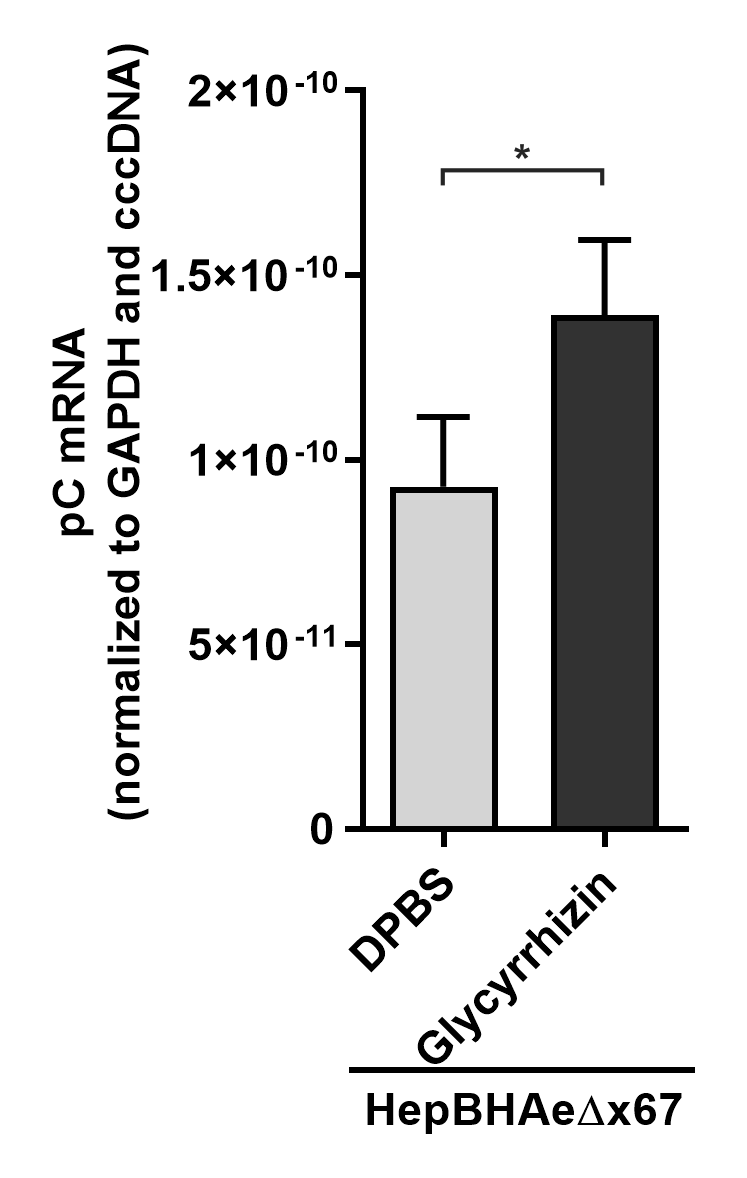

Supplement: S9 Fig — HepBHAeΔx67 cells were induced in tet-free medium for 8 days, followed by mock (1×DPBS) or glycyrrhizin (10 μM) treatment for 4 days in the presence of tet and 3TC (10 μM). HBV pC mRNA was quantified by RT-qPCR and normalized to GAPDH mRNA and cccDNA (mean ± SEM, n = 3), *p<0.05. (TIF) [file ppat.1010576.s009.tif]
