# Supplementary material for: Hepatitis B virus X protein counteracts high mobility group box 1 protein-mediated epigenetic silencing of covalently closed circular DNA
Source: PLoS Pathog. 2022 Jun 9;18(6):e1010576. doi: 10.1371/journal.ppat.1010576 (PMC9182688; doi:10.1371/journal.ppat.1010576)
Supplement: S1 Table — (PDF) [file ppat.1010576.s010.pdf]

S1 Table. Nuclear HBc pulldown host protein hits.

HBV cell lines	Gene names	Proteins names
(1) Both wt (HepAD38, tet-) and HBx-null (HepBHAeΔx67, tet-) HBV producing cell lines (non-histone proteins)	C18orf63	Uncharacterized protein C18orf63
	CEP126	Centrosomal protein of 126 kDa
	ITGA5	Integrin alpha-5
	NPM1	Nucleophosmin
	RPL4	60S ribosomal protein L4
	SRSF10	Serine/arginine-rich splicing factor 10
	TBC1D10C	Carabin
	UBB	Ubiquitin-60S ribosomal protein L40
(2) HepAD38 (tet-)	DLAT	Acetyltransferase component of pyruvate dehydrogenase complex
	HNRNPK	Heterogeneous nuclear ribonucleoprotein K
	URLC2	Bifunctional lysine-specific demethylase and histidyl-hydroxylase NO66
(3) HepBHAeΔx67 (tet-)	ACSL5	Long-chain-fatty-acid-CoA ligase 5
	BAG2	BAG family molecular chaperone regulator 2
	CLK3	Dual specificity protein kinase CLK3
	DDX17	Probable ATP-dependent RNA helicase DDX17
	DDX3X	ATP-dependent RNA helicase DDX3X
	DNAJB6	DnaJ homolog subfamily B member 6
	GAPDH	Glyceraldehyde-3-phosphate dehydrogenase
	GDF15	Growth/differentiation factor 15
	HMGB1	High mobility group box 1
	HNRNPA0	Heterogeneous nuclear ribonucleoprotein A0
	HSPA1A	Heat shock 70 kDa protein 1A and 1B
	LBR	Lamin-B receptor
	POR	NADPH-cytochrome P450 reductase
	PELP1	Proline-, glutamic acid- and leucine-rich protein 1
	PPIA	Peptidyl-prolyl cis-trans isomerase A
	RPS8	40S ribosomal protein S8
	SMAP	Small acidic protein
	SRSF1	Serine/arginine-rich splicing factor 1
	SRSF2	Serine/arginine-rich splicing factor 2
	SRSF6	Serine/arginine-rich splicing factor 6
	SRSF9	Serine/arginine-rich splicing factor 9
	THRAP3	Thyroid hormone receptor-associated protein 3
	VDAC2	Voltage-dependent anion-selective channel protein 2
	WDR18	WD repeat-containing protein 18
