# Supplementary material for: Hepatitis B virus X protein counteracts high mobility group box 1 protein-mediated epigenetic silencing of covalently closed circular DNA
Source: PLoS Pathog. 2022 Jun 9;18(6):e1010576. doi: 10.1371/journal.ppat.1010576 (PMC9182688; doi:10.1371/journal.ppat.1010576)
Supplement: S2 Table — (PDF) [file ppat.1010576.s011.pdf]

S2 Table. List of oligonucleotides used in the study.

target	oligonucleotide/primer sequences (5'→3')	
HBx C1397T (Gln8Stop) mutagenesis	Forward	CTGC**T**AACTGGATCCTGCGCGGGACGTCC (nt 1393-1421)
	Reverse	CACAGCCTAGCAGCCATGGATAC (nt 1392-1370)
HBx C1397T sequencing	Forward	TGGAGCAAACATTATCGGGA (nt 1313-1332)
	Reverse	AGTCCGCGTAAAGAGAGGTG (nt 1548-1529)
preCore mRNA RT-PCR	Forward	GTCTGCGCACCAGCACCAT (nt 1799-1817)
	Reverse	GTGCAGTATGGTGAGGTGAACAAT (nt 2042-2065)
β-actin mRNA RT-PCR	Forward	GCCGGGACCTGACTGACTACCTCAT
	Reverse	TTTGCGGTGGACGATGGAGG
Total HBV Hirt DNA qPCR	Forward	CCGTCTGTGCCTTCTCATCTG (nt 1551-1571)
	Reverse	AGTCCAAGAGTYCTCTTATGYAAGACCTT (nt 1674-1646)
	Probe	FAM- CCGTGTGCACTTCGCTTCACCTCTGC -TAMRA (nt 1577-1602)
cccDNA qPCR	Forward	TCATCTGCCGGACCGTGTGC (nt 1565-1584)
	Reverse	TCCCGATACAGAGCTGAGGCGG (nt 2021-2000)
	Probe	FAM-TTCAAGCCTCCAAGCTGTGCCTTGGGTGGC-TAMRA (nt 1865-1894)
preCore mRNA qPCR	Forward	GTAGGCATAAATTGGTCTG (nt 1785-1803)
	Reverse	GTGCAGTATGGTGAGGTGAACAAT (nt 2065-2042)
	Probe	FAM-CTCAGGAGACTCTAAGGCTTCCCGATACAG-TAMRA (nt 2040-2011)
COX3, mitochondrial	Forward	CCCTCTCGGCCCTCCTAATAACCTGC
	Reverse	GCCTTCTCGTATAACATCGCGTCA
GAPDH, exon 2-3	Forward	ACATCGCTCAGACACCATG
	Reverse	TGTAGTTGAGGTCAATGAAGGG
	Probe	FAM-AAGGTCGGAGTCAACGGATTTGGTC-TAMSp
