# Supplementary material for: Hepatitis B virus X protein counteracts high mobility group box 1 protein-mediated epigenetic silencing of covalently closed circular DNA
Source: PLoS Pathog. 2022 Jun 9;18(6):e1010576. doi: 10.1371/journal.ppat.1010576 (PMC9182688; doi:10.1371/journal.ppat.1010576)
Supplement: S3 Table — (PDF) [file ppat.1010576.s012.pdf]

S3 Table. List of antibodies used in the study.

Antibodies	Cat#, vendor	Dilution or amount per reaction
**Western Immunoblotting**		
Anti(α)-β-actin	4970S, Cell Signaling Technology	1:2,500
αHMGB1/HMG-1 (High Mobility Group Box 1)	MAB1690-100, R&D Systems, monoclonal mouse IgG2B	1:5,000
αHBx	1884, ViroStat	1:500
αFlag-tag	F3165, Sigma-Aldrich	1:1,000
αHA-tag (if HA-Co-IP)	3724S (C29F4), Cell Signaling Technology	1:1,000
IRDye® 800CW Goat α-mouse IgG	926-32210, Li-Core	1:10,000
IRDye® 800CW Goat α-rabbit IgG	926-32211, Li-Core	1:10,000
IRDye® 680RD Goat α-mouse IgG	926-68171, Li-Core	1:10,000
IRDye® 680RD Goat α-rabbit IgG	926-68172, Li-Core	1:10,000
**Immunofluorescence**		
α6×His-tag	SAB2702218-100UL, Sigma	1:1,000
αFLAG-tag	F3165, Sigma-Aldrich	1:1,000
Alexa Fluor 594 donkey α-mouse IgG (H+L)	A21203, Invitrogen	1:5,000
Alexa Fluor 594 goat α-rabbit IgG (H+L)	A11012, Invitrogen	1:5,000
Alexa Fluor 488 F(ab')2 fragment of goat α-mouse IgG (H+L)	A11017, Invitrogen	1:5,000
Alexa Fluor 488 F(ab')2 fragment of goat α-rabbit IgG (H+L)	A11070, Invitrogen	1:5,000
**Chromatin Immunoprecipitation for ChIP-qPCR**		
αH3K27ac (Histone H3 Lysine 27 acetylated)	b4729, abcam	5 µg
αH3K27me3 (Histone H3 Lysine 27 three-methylated)	ab6002, abcam	5 µg
αH3K4me3 (Histone H3 Lysine 4 three-methylated)	ab8580, abcam	5 µg
αH3K9me2 (Histone H3 Lysine 9 di-methylated)	A-4035, EpiGentek, polyclonal	5 µg
αH3K9me3 (Histone H3 Lysine 9 three-methylated)	ab8898, abcam	5 µg
αH4K20me2 (Histone H4 Lysine 20 di-methylated)	61533, Active Motif, monoclonal	5 µg
αHMGB1/HMG-1 (High Mobility Group Box 1)	MAB1690-100, R&D Systems, monoclonal mouse IgG_{2B}	5 µg
αSmc6 (Structural maintenance of chromosomes 6)	sc-365742-X (A-3), Santa Cruz, monoclonal	5 µg

αHistone H1	A68325, EpiGentek, polyclonal	5 µg
αSetDB1/ESET (SET domain bifurcated 1; KAP-1-associated histone H3, lysine 9-specific methyltransferase)	07-1568, Millipore Sigma, polyclonal	5 µg
αSetDB2 (SET domain bifurcated 2; Histone-lysine N-methyltransferase)	05-1952, clone 5B7.2 Millipore Sigma, monoclonal	5 µg
αPan α-crotonyl lysine	PTM-501, PTM Biolabs	5 µg
αRNAP II CTD repeat YSPTYSPS (phospho S5) [4H8]	ab5408, ChIP grade, abcam	5 µg
IgG, non-immune serum (NIS) control	I8765-5MG IgG from mouse serum, Sigma	5 µg
**Proteins-cccDNA complex immunoprecipitation for mass-spectrometry**		
αHBc	B0586, Dako	1:50
**CLIA/ELISA**		
αHA-Tag Antibody, mAb, Mouse	A01244, Genscript	5 µg/ml in PBS buffer
α-HBeAg-HRP	61-H10K, clone M10071922, Fitzgerald	1: 40,000
